# Trends in vaccination coverage and equity in the Democratic Republic of the Congo from 2017 to 2023

**DOI:** 10.1016/j.vaccine.2025.127609

**Published:** 2025-08-30

**Authors:** Elise Lankiewicz, Junias Kabele Ngoy Mpemba, Paul Samson Lusamba Dikassa, Viviane Mayala Masiala, Benito Kazenza Maykondo, Trad Hatton, Saira Nawaz, Wolfgang Munar, Catherine Arsenault

**Affiliations:** a Department of Global Health, The George Washington University Milken Institute School of Public Health, Washington, DC, United States; b Department of Environmental Health, School of Public Health, University of Kinshasa, Kinshasa, Democratic Republic of the Congo; c Department of Epidemiology and Biostatistics, School of Public Health, University of Kinshasa, Kinshasa, Democratic Republic of the Congo; d Department of Health Policy and Management, School of Public Health, University of Kinshasa, Kinshasa, Democratic Republic of the Congo; e Department of Nutrition, School of Public Health, University of Kinshasa, Kinshasa, Democratic Republic of the Congo; f PATH, Kinshasa, Democratic Republic of the Congo; g PATH, Washington, DC, United States

**Keywords:** Equity, Coverage, Routine immunization strengthening

## Abstract

**Introduction:**

Several routine immunization (RI) strengthening efforts have been implemented in the Democratic Republic of the Congo (DRC) in the last decade. However, there has been no assessment of national or provincial-level trends in inequalities in RI coverage since the implementation of these programs. In this analysis, we aimed to describe trends in childhood vaccination coverage and inequalities from 2017 to 2023 at the national and provincial levels and to compare these trends among groups of provinces where two initiatives have been in place: the Mashako plan and a provincial level public-private partnership using a memorandum of understanding (MOU) approach.

**Materials and methods:**

We used population-based surveys including the Multiple Indicator Cluster Survey (MICS) – Palu 2017–2018 survey and four annual vaccination coverage surveys conducted from 2020 through 2023. We described vaccination coverage (three doses of pentavalent vaccine (Penta3) and at least one dose of a measles containing vaccine (MCV1)) and assessed relative and absolute inequalities in vaccination coverage by maternal education and household wealth at each time point. Analyses were conducted at the national level and within two groups of provinces: those initially included in the Mashako plan in 2018 and those initially included in the MOU approach. Inequality estimates were pooled across province groups using a random effects DerSimonian and Laird estimator for meta-analysis.

**Results:**

From 2017 to 2023, national Penta3 coverage increased by 9.9 percentage points (47.7 % to 57.6 %) while MCV1 declined by 6.7 percentage points (58.9 % to 52.2 %). As of 2023, substantial wealth and education-related inequalities in childhood vaccination coverage remained: at the national level, children from wealthier households were 2.23 times more likely to receive Penta3 compared to children from poorest households (95 % Confidence Interval (CI) 2.16–2.31). Between 2017 and 2023, absolute and relative wealth-related inequalities appear to have declined, but differences were not statistically significant. Education-related inequalities have improved less than wealth-related inequalities. Though differences were often not statistically significant, reductions in inequalities were generally larger in provinces initially included in the Mashako plan and the MOU approach than in provinces not initially included in either initiative. Initial improvements in coverage and inequality between 2017 and 2020–2021 have largely stagnated at the national and sub-national levels in 2022 and 2023.

**Discussion:**

Efforts remain needed to reach RI coverage and equity targets in the DRC. Routine monitoring of inequalities in RI coverage should be performed regularly to track progress. A more explicit equity focus in RI strengthening initiatives in the DRC may be necessary to accelerate progress in reducing existing inequalities.

## 1 Introduction

Routine immunization (RI) is one of the most cost-effective interventions available for reducing child morbidity and mortality, yet approximately 20 million infants globally do not receive a full course of basic vaccines annually [1]. In the Democratic Republic of the Congo (DRC), a country with nearly 20 million children under the age of 5, substantial improvements in vaccination coverage since 2000 had stalled or eroded by the mid-2010s, resulting in several measles and vaccine-derived poliovirus outbreaks [[2], [3], [4]]. Challenges to sustained RI coverage in the DRC are multi-faceted. The country is spread over nearly 2.3 million km^2^, with critical limitations in health infrastructure furthered by incomplete decentralization, civil unrest, and armed conflict [5,6]. Poverty remains widespread with more than 80 % of the rural population in DRC living below the international poverty line, and outside of mineral rich areas and the capital, many provinces have slim revenue generation prospects and similarly low health budgets [5,7,8]. Competing outbreaks in the context of limited resources and vaccine hesitancy similarly complicate vaccination efforts [[9], [10], [11]].

In 2018, in response to dropping vaccination rates and a series of vaccine-preventable disease outbreaks, the DRC Expanded Program on Immunization (EPI) launched an emergency response program termed the “Mashako Plan” in nine high-priority provinces: Kinshasa, Haut Katanga, Kwilu, Kasai, Ituri, Mongala, Tshuapa, Tanganyika, and Haut Lomami [12]. The effort proposed to improve coverage through strengthened monitoring and evaluation, coordination, service delivery, and vaccine availability [12]. Descriptive assessments of the initial implementation of the Mashako plan found greater improvement in RI coverage and process indicators in Mashako provinces relative to non-Mashako provinces – though COVID-related disruptions represented a challenge nationally in 2020 onward [12]. In 2020, the initial coverage improvements prompted the government to expand the Mashako plan to the remaining provinces the following year, though roll-out was delayed by the COVID pandemic [12].

During the implementation of the Mashako plan, support was provided by a variety of funders and technical partners including the Bill and Melinda Gates Foundation (BMGF) and Gavi [12]. At the beginning of the Mashako implementation in 2018, BMGF signed a 51-month Memorandum of Understanding (MOU) with the provincial governments of two of the initial Mashako plan provinces: Haut Lomami and Tanganyika [13]. These provinces were prioritized given their lower immunization coverage and reemergence of circulating vaccine-derived poliovirus [13]. Both Haut Lomami and Tanganyika present challenging contexts for RI, with large proportions of rural and hard-to-reach populations as well as ongoing conflict in Tanganyika [14,15].

The MOU approach in DRC was based on a similar intervention implemented by BMGF and partners in six northern states in Nigeria between 2012 and 2022 [16]. The Nigerian MOUs resulted in some substantive gains in vaccination coverage, however, the extent of progress in coverage varied by state, and the impacts on health systems strengthening were more limited [17]. The MOUs in the DRC similarly aimed to increase vaccination coverage through provincial empowerment, provincial financial contributions to RI, improved accountability to RI targets, and expertise transfer to Provincial Divisions of Health [13]. In addition to a shared basket-fund paid into by BMGF and provincial governments, initial implementation of the MOUs included capital investments in cold chains and transport, establishing coordination structures, the use of new microplanning strategies, and extensive Technical Assistance (TA) provision [13]. During early implementation, Haut Lomami, Tanganyika, and Lualaba (a third province that signed an MOU in 2021) all saw improvements in vaccination coverage by 2022, though provincial-level results varied, and gaps in financial management capabilities and contributions persisted [13].

While these early data point to potential improvements in coverage in Mashako and MOU provinces, to the best of our knowledge, there has been no assessment of national or provincial-level trends in equity in RI coverage during the implementation period. Equity in RI coverage refers to differences in vaccination rates between population groups – characterized by factors like socioeconomic status, education, geography, ethnicity or gender – which are unnecessary, avoidable and unjust [18]. Operationalizing the measurement of inequities often involves monitoring health inequalities, or systematic differences, among potentially vulnerable populations. Monitoring inequalities in health services is critical to ensuring universal access to services, especially given that programming without explicit equity approaches can lead to widened inequalities as most advantaged groups tend to be the easiest to reach [19,20]. Assessment of DRC vaccination coverage based on the 2013 Demographic Health Survey pointed to substantial levels of inequality by key characteristics, including household wealth and maternal education [21]. In this paper, we describe trends in vaccination coverage and inequalities in vaccination coverage from 2017 to 2023 in the DRC. We also compare coverage and inequality in groups of provinces included in two immunization efforts, the Mashako plan and the BMGF MOU approach, over the six-year period.

## 2 Material and methods

### 2.1 Data sources

For baseline data before the implementation of the Mashako Plan and the MOUs, we used data from the Multiple Indicator Cluster Survey (MICS) – Palu conducted in 2017–2018 [22]. In the DRC, MICS surveys are representative at the provincial level and include demographic and health-related information, including vaccination coverage [22]. Data from 2020 to 2023 were drawn from four annual vaccination coverage surveys (Enquête de Couverture Vaccinale [ECV]), which also include demographic information and vaccination coverage estimates. In the DRC, there are six levels of the health system playing different roles in RI (national, provincial, antenne, health zone, health area, and health facility). ECVs are representative at the provincial and health zone-level [[23], [24], [25], [26]]. ECV surveys conducted in 2021, 2022 and 2023, were conducted in all 26 provinces while the 2020 ECV included only 18 out of 26 provinces. ECVs used a multi-stage stratified cluster sampling approach. In each province, all health zones (*N* = 519 across 26 provinces) were surveyed. Sampling in each health zone was conducted in three stages: first, health areas were randomly selected (about five per health zone); next, segments—covering roughly 30 % of villages in rural areas or 30 % of streets in urban areas—were randomly chosen; finally, households within those segments were randomly selected (approximately 30 per village or street). Further details on the sampling strategies and methodologies for MICS and ECV surveys are available eleshwere [[22], [23], [24], [25],^27^].

### 2.2 Measures

The study assessed receipt of the third dose of the pentavalent DTwP-HepB-Hib vaccine (Penta3), one dose of a measles-containing vaccine (MCV) and full vaccination with eight basic vaccines (Bacille Calmette–Guérin, three doses of an oral polio vaccine, Penta3, and MCV) among children 12–23 months [28]. Penta3 and MCV are part of the DRC's routine immunization schedule, and are both often used as sentinel indicators to assess a country's immunization program strength [12,29]. In MICS and ECV, vaccination data were collected from both caregiver recall and assessment of vaccination cards [[22], [23], [24], [25]]. In this analysis, a child was counted as having received a vaccine if vaccination was reported from either source.

Wealth indices, gender, and maternal educational attainment were used in inequality calculations. Wealth and education have been identified as underlying socioeconomic factors distally related to child health [30]. The MICS wealth index utilizes a methodology developed by Rutstein et al. (2004, 2008, 2014) and used by the Demographic and Health Surveys (DHS), in which principal component analysis (PCA) is run on a set of household assets and characteristic variables to optimize the inclusion of variables assumed to best represent wealth [[31], [32], [33], [34]]. For all ECV surveys, the steps for constructing the DHS wealth index were followed, with the notable difference that ECV surveys do not include household infrastructure-related variables to be considered for inclusion in index construction and accordingly only included household asset variables. For ECV 2020, no indicator for urban and rural households was included in the survey, so a single common wealth index was created. For ECV 2021, ECV 2022, and ECV 2023, separate indices were developed for rural and urban regions and then scaled to a single wealth distribution following DHS methodology, allowing for comparability of wealth scores across urban and rural groups [32]. An ordinal variable for maternal education was used, with levels coded for no, primary, secondary, and above secondary education.

### 2.3 Province groups

We created two groups of provinces. The first included the nine provinces that were in the initial implementation of the Mashako plan in 2018: Kinshasa, Haut Katanga, Kwilu, Kasai, Ituri, Mongala, Tshuapa, Tanganyika, and Haut Lomami [12]. The other 17 provinces not initially included served as a comparison group. For our analysis of the initial implementation of the Mashako plan, we compare 2017 (pre-Mashako implementation) to the ECV post implementation of the first Mashako plan (‘Mashako 1.0) in 2021 [12]. While a second iteration of the Mashako plan was implemented, our analysis focuses only on the initial implementation. We also compared the two provinces part of the MOU approach (Haut-Lomami and Tanganyika) to the remaining seven provinces included in the initial Mashako plan but not part of the MOU approach (Kinshasa, Kwilu, Kasai, Ituri, Mongala, Tshuapa, and Haut Katanga). While an MOU was signed in Lualaba province in 2021, this analysis focuses on the initial MOU implementation, and accordingly, Lualaba is not included in the MOU grouping [13]. For our analysis of the initial implementation of the MOU approach, we compare 2017 (pre-MOU) to 2022, the ECV post the end line of the initial MOU (MOU 1.0) [13]. Similar to the Mashako plan, while a second round of MOUs were later implemented, our analysis focuses only on the initial round of the MOU approach.

### 2.4 Statistical analysis

We first described Penta3 and MCV coverage at the national level and in the province groups across the five survey years. To assess differences in vaccination coverage by gender, we used logistic regression and post-estimation to generate risk differences [35]. For wealth- and education-related inequalities, we assessed relative inequalities in vaccination coverage using the relative index of inequality (RII) and absolute inequalities using the slope index of inequality (SII). It is recommended that both relative and absolute measures of inequality be presented to assess both proportional differences and magnitude [19,36]. RII and SII describe inequality across the entire gradient of the socioeconomic distribution, in contrast with other absolute measures of inequality that only compare extreme ends of the socioeconomic measures [19,21,36]. To calculate the SII and RII, individuals were ordered according to their wealth index score or maternal education level within provinces and assigned a rank in accordance with their position across the distribution [21]. Logistic regression was used to estimate the association between relative rank for wealth or education and vaccination coverage for each vaccine of interest, followed by post-estimation of RII and SII [21].

After RII and SII were calculated for each province, we pooled the SII and log-transformed and back-transformed RII in the initial Mashako provinces and non-Mashako provinces by inverse variance weighted random effects meta-analysis using the DerSimonian and Laird method [37]. A simple difference was used to assess the change in RII and SII, comparing pre-Mashako 1.0 implementation (2017) and at the end of the Mashako 1.0 period (2021). We also pooled the SII and RII in the two provinces part of the MOU approach, Haut Lomami and Tanganyika, and compared estimates to those in the remaining seven non-MOU Mashako 1.0 provinces. Estimated changes in relative and absolute inequality were calculated as the difference in RII or SII between 2022 (the end of the initial MOU period) and 2017 (pre-MOU) [38].

Analyses were conducted in Stata 18.0, and R Studio 2024.04.2 [39,40]. Sampling weights were used in all analyses. This study used secondary de-identified data and, accordingly, was exempt from Institutional Review Board review.

## 3 Results

### 3.1 Coverage

**Fig. 1:**
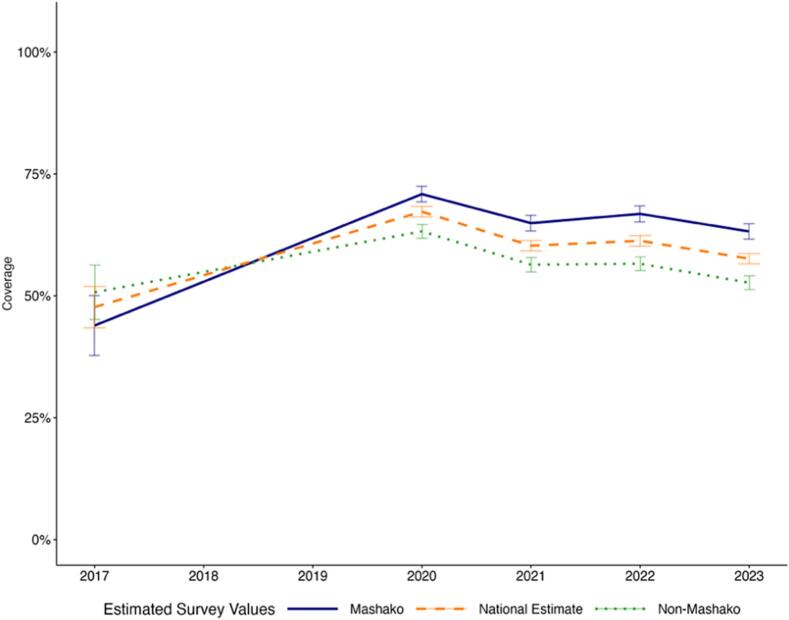

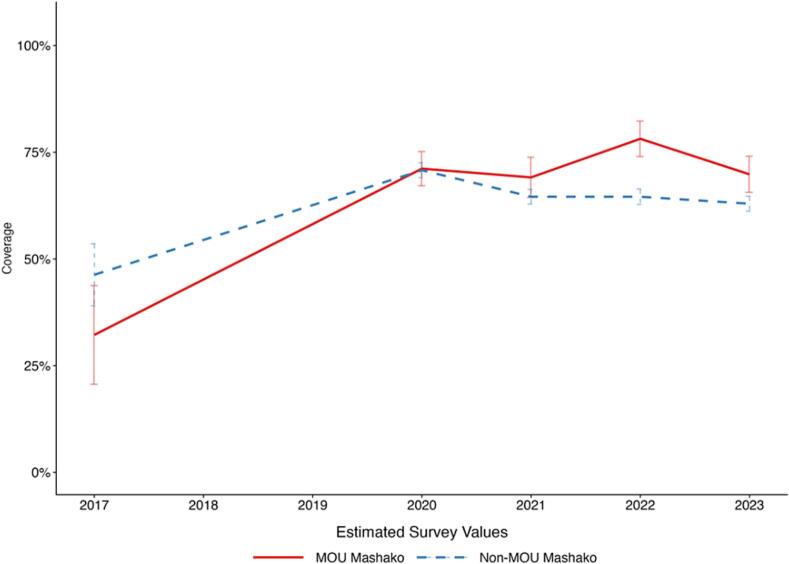

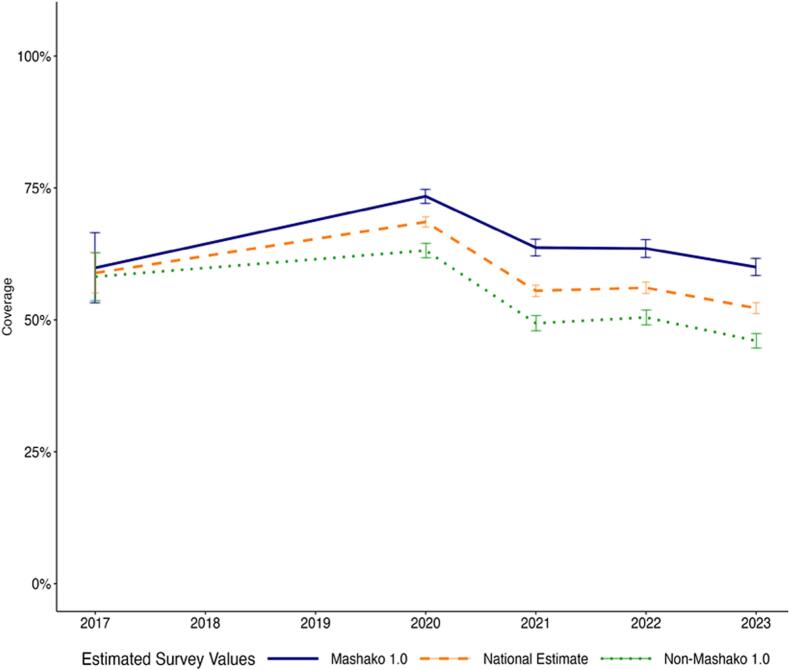

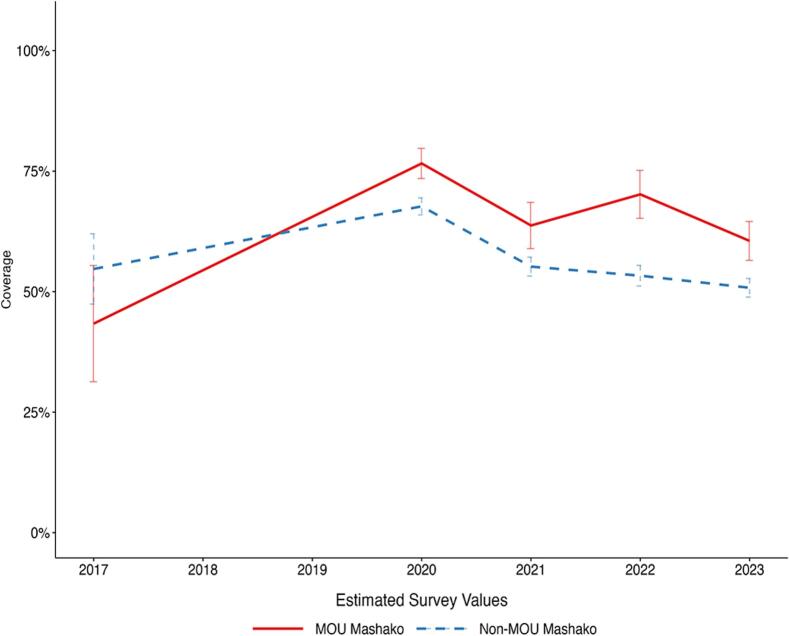
A. Penta3 coverage estimates at the national level and in Mashako 1.0 and non-Mashako 1.0 provinces from 2017 to 2023. B. Penta3 coverage estimates within Mashako 1.0 provinces in MOU 1.0 and non-MOU 1.0 provinces from 2017 to 2023. C. MCV coverage estimates at the national level and in Mashako 1.0 and non-Mashako 1.0 provinces from 2017 to 2023. D. MCV coverage estimates within Mashako 1.0 provinces in MOU 1.0 and non-MOU 1.0 provinces from 2017 to 2023.

Between 2017 and 2023, Penta3 coverage increased from 47.68 % to 57.61 % at the national level, an increase of 9.93 percentage points (Fig. 1A). During implementation of the initial Mashako plan, comparing 2017 to 2021 Penta3 coverage in the initial Mashako plan provinces increased more than in non-Mashako provinces. In the Mashako provinces, coverage increased from 44.62 % in 2017 to 65.43 % in 2021, a 20.81 percentage-point increase. In the non-Mashako provinces, coverage increased from 49.83 % to 56.48 % over the same period, a smaller 8.65 percentage point increase. Within the Mashako provinces comparing 2017 to the endline of initial implementation in 2022, coverage in the two MOU provinces improved by a substantive 45.95 percentage points to 78.15 % while the non-MOU Mashako provinces improved by 18.29 percentage points to 64.58 % (Fig. 1B). Notably, estimated coverage was lower in all groups (National, Mashako, Mashako MOU, and non-MOU Mashako), in 2023 as compared to 2022. In contrast with Penta3, national-level MCV coverage decreased between 2017 and 2023, from 58.87 % to 52.22 %. Across province groups, only the MOU 1.0 province group, saw an increase in MCV coverage from 43.37 % in 2017 to 60.53 % in 2023 (Fig. 1C**-**D).

### 3.2 Inequalities in RI coverage

#### 3.2.1 National-level inequalities

**Fig. 2:**
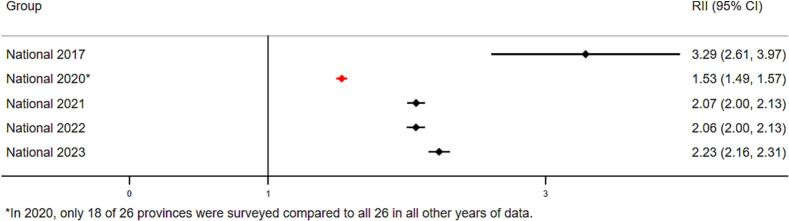

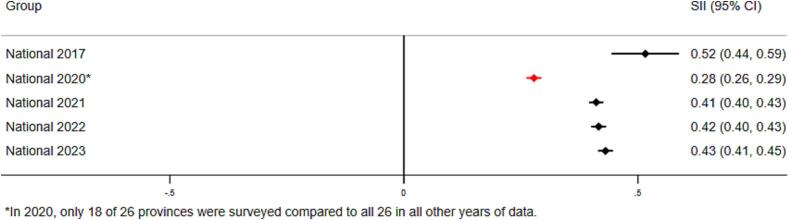

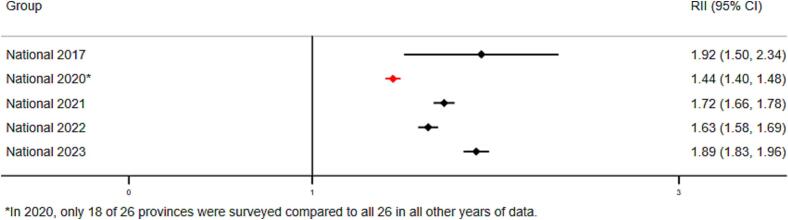

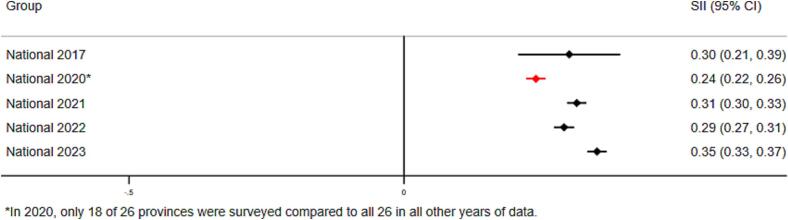
A. National-level wealth-related relative index of inequality (RII) in Penta3 vaccination (2017–2023). B. National-level wealth-related slope index of inequality (SII) in Penta3 vaccination (2017–2023). C. National-level education-related relative index of inequality (RII) in Penta3 vaccination (2017–2023). D. National-level education-related slope index of inequality (SII) in Penta3 vaccination (2017–2023).

Virtually no differences were found between boys and girls in any survey year. We also assessed inequality for full vaccine coverage, but present only MCV and Penta3, given full vaccination trends were qualitatively similar to that of Penta3. Fig. 2 presents absolute and relative wealth- and education-related inequalities in Penta3 coverage at the national level. Large and statistically significant inequalities in Penta3 vaccination coverage persist as of 2023 (Fig. 2A**-**D). In 2023, the national RII for wealth was 2.23 (CI 2.16–2.31), suggesting that, on average, the children from wealthiest households are 2.23 times more likely to be vaccinated as compared to children from poorest households in DRC. This decreased from an RII of 3.29 (CI 2.61–3.97) in 2017. The SII for wealth was 0.43 (CI 0.41–0.45) in 2023, suggesting that the proportion of vaccinated children is 43 percentage points higher on average among children at the top of the wealth distribution compared to the bottom. This was an improvement from the 2017 SII of 0.52 (CI 0.44–0.59).

Inequalities related to maternal education levels also remain as of 2023, but in contrast to wealth-related inequalities, they have changed minimally since 2017 (Fig. 2C-D). Children of most educated mothers were 1.89 times more likely to have received Penta3 compared to children of least educated mothers (RII = 1.89, 95 % CI 1.83–1.96). Absolute education-related inequalities have increased at the national level between 2017 and 2023, though this increase is not statistically significant. The magnitude and trend in inequalities in MCV coverage at the national level were similar to those for Penta 3 and are shown in **supplemental materials**.

#### 3.2.2 Inequalities among Mashako and non-Mashako provinces

**Fig. 3:**
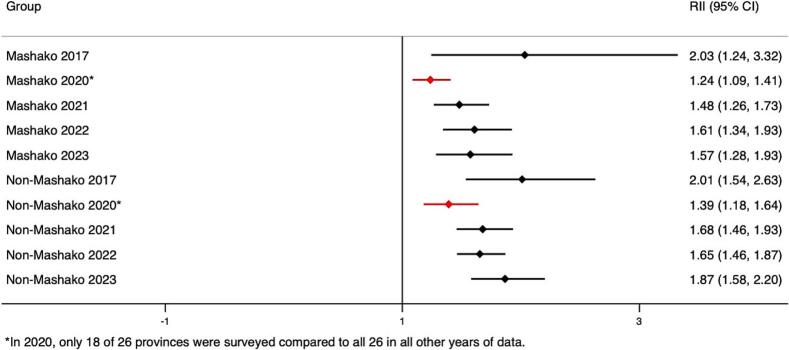

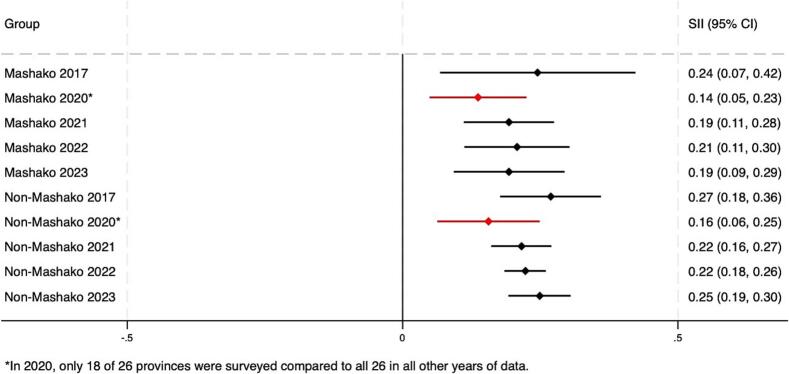

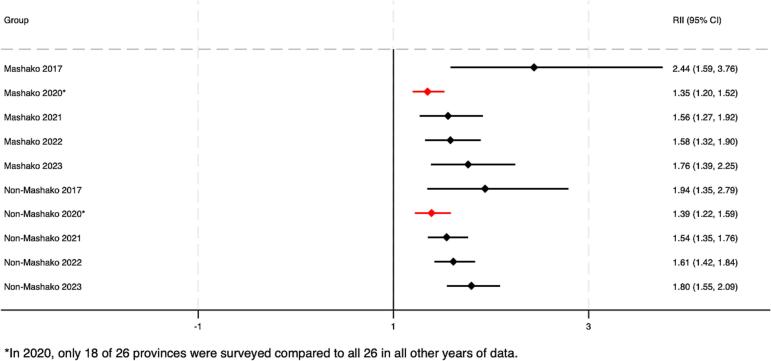

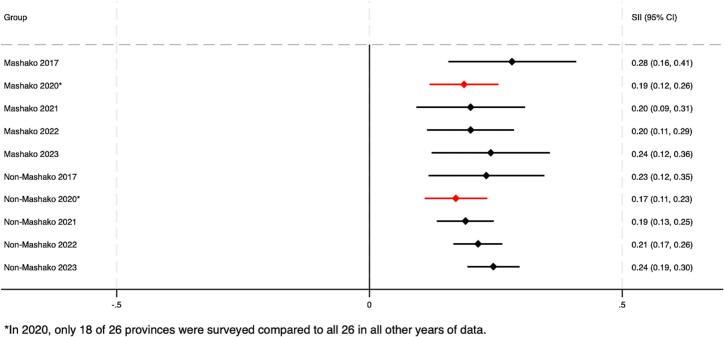
A. Pooled wealth-related relative index of inequality (RII) in Penta3 vaccination (2017–2023) in Mashako 1.0^&^ vs. non-Mashako provinces. B. Pooled wealth-related slope index of inequality (SII) in Penta3 vaccination (2017–2023) in Mashako 1.0^&^ vs. Non-Mashako provinces. C. Pooled education-related relative index of inequality (RII) in Penta3 vaccination (2017–2023) in Mashako 1.0^&^ vs. Non-Mashako provinces. D. Pooled educated-related slope index of inequality (SII) in Penta3 vaccination according (2017–2023) in Mashako 1.0^&^ vs. Non-Mashako provinces.

As of the end of Mashako 1.0 in 2021, pooled estimates reveal that statistically significant wealth- and education-related relative and absolute inequality in Penta 3 coverage remained in non-Mashako and Mashako province groups (Fig. 3A-D). Levels of inequality between Mashako and non-Mashako province groups were similar at endline (2021). We assessed changes over time and found that all groups saw non-statistically significant improvements in wealth-related inequalities (Table 1). Relative inequality decreased more over this period in the Mashako group compared to the non-Mashako group, though this difference was not statistically significant either. Both groups saw similar improvements in relative and absolute educational inequalities, and the improvement in educational inequality was larger (though not statistically significant) in the Mashako group compared to the non-Mashako group. Inequality trends were similar for MCV. However, unlike for Penta 3, the absolute (SII) inequality reductions between 2017 and 2021 were statistically significant in both Mashako and non-Mashako groups (see **supplemental materials**).

**Table 1:** Change in Penta3 inequality during the Mashako 1.0^a^ period 2017–2021 (95 % CI).

	RII 2017 (CI)	RII 2021 (CI)	RII Change (CI)	SII 2017 (CI)	SII 2021 (CI)	SII Change (CI)
Wealth						
Mashako	2.03 (3.32, 1.24)	1.48 (1.73, 1.26)	−0.55 (−1.62, 0.51)	0.24 (0.42, 0.07)	0.19 (0.28, 0.11)	−0.05 (−0.25, 0.14)
Non-Mashako	2.01 (2.63, 1.54)	1.68 (1.93, 1.46)	−0.33 (−0.93, 0.26)	0.27 (0.36, 0.18)	0.22 (0.27, 0.16)	−0.05 (−0.16, 0.05)
Difference, Mashako - Non-Mashako			−0.22 (−1.44, 1.00)			0.00 (−0.22, 0.22)

Education						
Mashako	2.44 (3.76, 1.59)	1.56 (1.92, 1.27)	−0.88 (−2.02, 0.25)	0.28 (0.41, 0.16)	0.2 (0.31, 0.09)	−0.08 (−0.25, 0.08)
Non-Mashako	1.94 (2.79, 1.35)	1.54 (1.76, 1.35)	−0.39 (−1.15, 0.36)	0.23 (0.35, 0.12)	0.19 (0.25, 0.13)	−0.04 (−0.17, 0.09)
Difference, Mashako - Non-Mashako			−0.49 (−1.85, 0.87)			−0.04 (−0.25, 0.17)

^a^Mashako provinces include those included in the original implementation of the Mashako Plan 1.0: Mongala, Tshuapa, Haut Katanga, Ituri, Kinshasa, Kwilu, and Kasaï. The non-Mashako group includes all other provinces in the country.

#### 3.2.3 Inequalities among MOU and non-MOU provinces

**Fig. 4:**
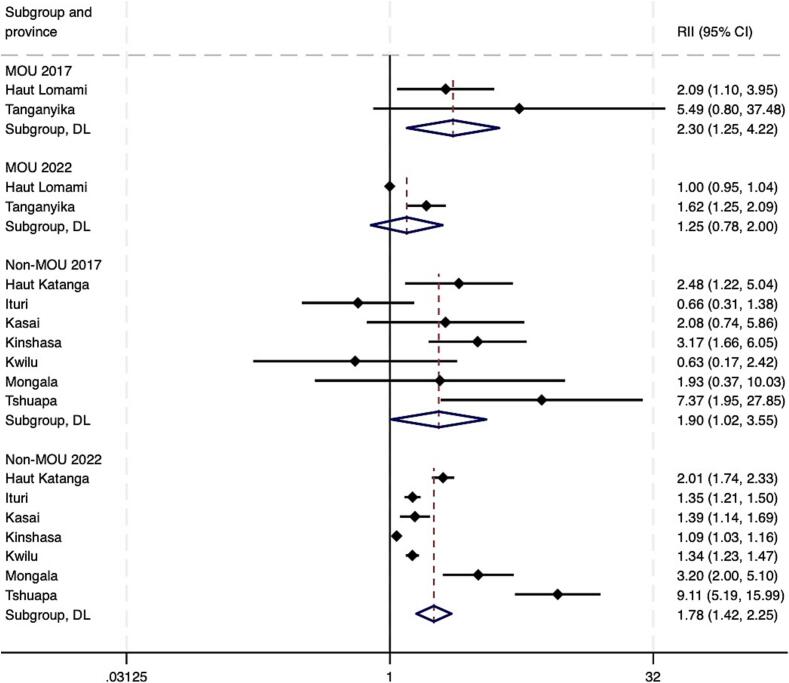

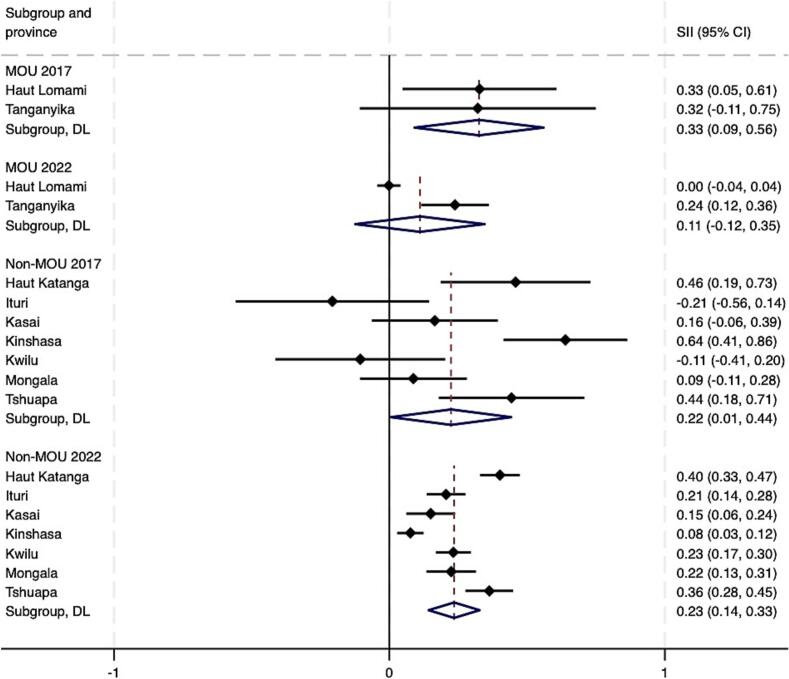

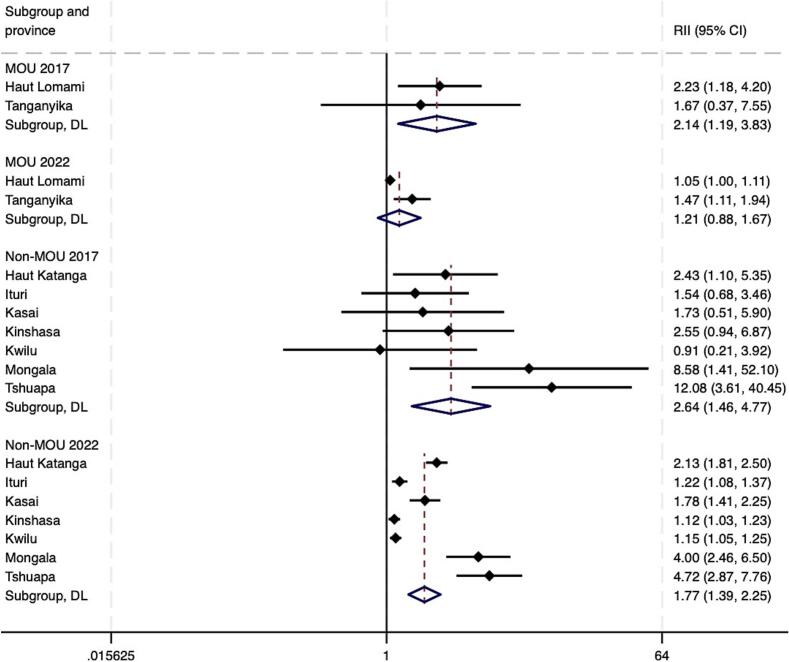

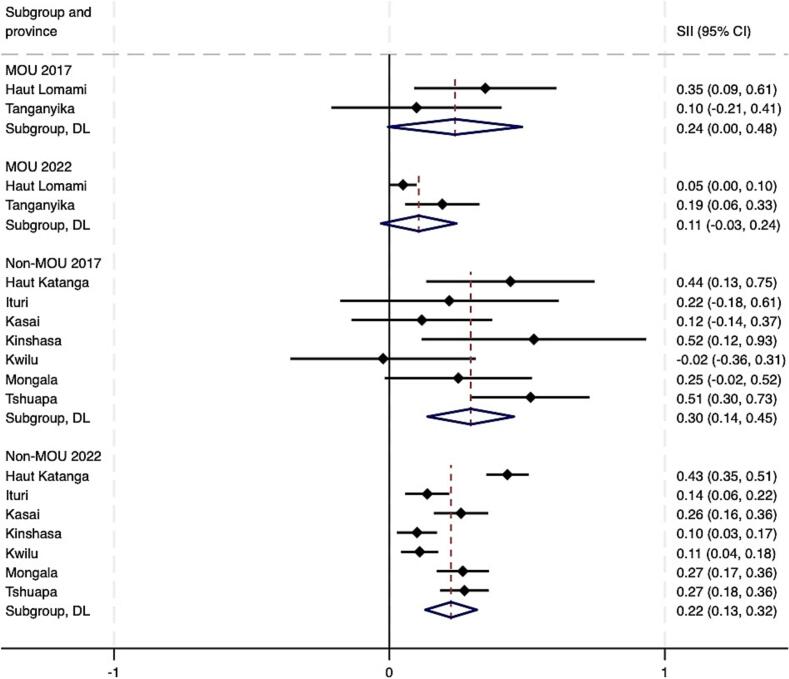
A. Wealth-related relative index of inequality (RII) in Penta3 vaccination, pre-MOU (2017) and endline-MOU 1.0 (2022). B. Wealth-related slope index of inequality (SII) in Penta3 vaccination, pre-MOU 1.0 (2017) and endline-MOU 1.0 (2022). C. Education-related relative index of inequality (RII) in Penta3, pre-MOU 1.0 (2017) and endline-MOU 1.0 (2022). D. Education-related slope index of inequality (SII) in Penta3, pre-MOU 1.0 (2017) and endline-MOU 1.0 (2022).

As of 2022, pooled estimates suggest that both absolute and relative wealth-related inequalities in Penta 3 coverage remained in both MOU and non-MOU Mashako provinces (Figs. 4A – 4B). At the provincial level, by 2022, virtually no wealth-related absolute or relative inequalities were present in Haut Lomami (RII = 0.00, 95 % CI -0.04 - 0.04; SII = 1.05, 95 % CI 1.00–1.00). Improvements were larger in the MOU group as compared to the non-MOU group, though these differences were not statistically significant (Table 2). Trends among the non-MOU group varied, with non-uniformity in whether provincial-level inequalities worsened or improved by the end of MOU 1.0 implementation in 2022. When assessing pooled estimates, groups saw similar improvements in education-related relative and absolute inequalities. The MOU group improved relative inequality during initial implementation by 0.05 more than the non-MOU group, and absolute educational inequality by 0.06 more than the non-MOU group. While trends were relatively similar for MCV, the improvement in absolute wealth- and education-related inequalities in MCV were greater and statistically significant in the MOU group compared to the non-MOU province group. This was also true for improvements in relative educational inequalities in MCV coverage (see **supplemental materials**).

**Table 2:** Change in Penta3 inequality during the MOU 1.0^a^ period 2017–2022 (95 % CI).

	RII 2017 (CI)	RII 2022 (CI)	RII Change (CI)	SII 2017 (CI)	SII 2022 (CI)	SII Change (CI)
Wealth						
MOU	2.30 (4.22, 1.25)	1.25 (2.00, 0.78)	−1.05 (−2.66, 0.56)	0.32 (0.56, 0.09)	0.11 (0.34, −0.12)	−0.21 (−0.54, 0.12)
Non-MOU	1.90 (3.55, 1.02)	1.78 (2.24, 1.42)	−0.12 (−1.43, 1.19)	0.22 (0.44, 0.01)	0.23 (0.33, 0.14)	0.01 (−0.23, 0.25)
*Difference, MOU - Non-MOU*			−0.93 (−3.01, 1.15)			−0.22 (−0.63, 0.18)

Education						
MOU	2.13 (3.83, 1.19)	1.21 (1.67, 0.88)	−0.92 (−2.29, 0.45)	0.24 (0.48, −0.00)	0.11 (0.24, −0.03)	−0.13 (−0.40, 0.14)
Non-MOU	2.64 (4.77, 1.47)	1.77 (2.25, 1.39)	−0.87 (−2.58, 0.84)	0.30 (0.45, 0.14)	0.22 (0.32, 0.13)	−0.07 (−0.25, 0.11)
*Difference, MOU - Non-MOU*			−0.05 (−2.25, 2.14)			−0.06 (−0.39, 0.27)

^a^The MOU group includes Haut Lomami and Tanganyika. The non-MOU group includes Haut Katanga, Ituri, Kasaï, Kinshasa, Kwilu, Mongala, and Tshuapa.

## 4 Discussion

In this paper, we used nationally representative RI surveys from the DRC to estimate coverage and inequalities during the implementation of programs aimed at improving RI in the DRC from 2017 to 2023. At the national-level, substantial inequalities in Penta3 coverage remain according to both maternal education and household wealth. Improvements in coverage and equity have largely stalled from 2021 onwards. Provinces where the Mashako Plan and BMGF MOUs were implemented generally saw greater improvements in coverage and equity than in provinces without either program, although differences were often not statistically significant.

At the national level, improvements in wealth-related inequalities were not matched by similar progress in education-related inequalities. Maternal education has been consistently found to be strongly associated with child health decision-making across contexts and may capture a critical aspect of gender-related social exclusion [21,[41], [42], [43], [44], [45]]. Lu et al. suggest that maternal educational attainment allows for improved health literacy and the ability to communicate with healthcare providers, leading to higher demand for vaccination [44]. This may be particularly important in the DRC context, where Ishoso et al. found strong associations between parental perceptions and feelings towards vaccines and zero-dose status, referring to infants who have never received any dose of a diphtheria-pertussis–tetanus-containing vaccine [11]. The differing trends in wealth and maternal education-related inequalities in our study reaffirm the importance of tracking multiple measures related to socioeconomic inequalities. Notably, coverage improvements in Penta3 have outpaced those for MCV which has decreased nationally since 2017. Since MCV is generally given later than Penta3 (at 9–11 months old), efforts aimed at reducing vaccination drop-out remain critical [3,46].

Among Mashako and MOU provinces and province groups, pooled inequality estimates either remained stable or improved following initial program implementation. While improved coverage often aligns with reduced inequality, this is not always the case, especially for routine-facility-based vaccination, where higher socioeconomic groups may have easier access to facilities (thus potentially widening inequalities) [47]. This could be particularly true in the DRC, where getting vaccines to poor rural areas can be exceptionally challenging: vaccine doses arrive through Kinshasa and many rural locations are unreachable by road, requiring river-based transport [15,48]. Outreach campaigns must also reach highly mobile populations moving as a result of conflict, artisanal mining, and subsistence farming. [49,50] Accordingly, given the strong relationship between wealth and urbanicity in the DRC, interventions that directly target remote populations may be expected to have a substantive impact on wealth-related inequalities. In the two MOU provinces we assessed (Haut-Lomami and Tanganyika), we found large improvements in wealth-related inequalities in the initial years of implementation. MOU-related investments in RI infrastructure that are particularly relevant to reaching remote populations may help explain this improvement. MOU provinces invested heavily in cars, motorcycles, outboard motors, portable vaccine coolers and solar power refrigerators, likely expanding the reach of vaccination to rural populations [13]. Resilient cold chains in remote contexts have been previously identified as important for equitable vaccine access [51,52].

Assessment of the MOU provinces compared to the other non-MOU Mashako provinces shows distinct non-uniformity in trends at the provincial level. This corresponds with other DRC-vaccination analyses that find vast differences across provinces, reinforcing the importance of sub-national analyses to identify trends in inequality that may not be apparent in national-level measures [11,53]. Among MOU provinces, Haut Lomami had eliminated wealth-related inequalities by the end of the MOU 1.0 implementation in 2022. Existing assessments of the early impact of the MOU 1.0 similarly found that Haut Lomami outperformed Tanganyika on MOU approach process indicators such as vaccine availability, though both provinces struggled to meet targets for provincial financial contributions to RI activities [13]. Smaller improvements in coverage and inequality in Tanganyika align with previous work that cites the COVID-19 pandemic and nursing strikes as differentially impacting Tanganyika relative to Haut Lomami [13]. Overall, initial improvements observed in 2020–21 appear to have slowed in 2022–23. It is unclear whether these setbacks are entirely due to declines in coverage due to increased political instability in the DRC, post-pandemic effects or funding reductions. They may also be partly due to methodological artifacts from sampling changes in 2023 as explained further below.

Other studies have described the design and process outcomes related to the Mashako Plan and BMGF MOUs in DRC as well as changes in vaccination coverage through the ECV surveys [12,13,27]. Maternal education and wealth-related vaccination inequalities at the national level have also been assessed in the DRC using the 2013 Demographic and Health Survey (DHS) [21]. To the best of our knowledge, however, our paper is the first to report coverage estimates and inequalities at the provincial level during the implementation of the Mashako Plan and MOUs. Strengths of our analysis include the use of five time points and that we assessed inequality at the sub-national level. Sub-national analyses are particularly important in the DRC, given the vastly different contexts and existing trends in vaccination coverage, poverty, and educational disparities in different provinces [5,11,54]. Even within Mashako provinces, some like Kinshasa and Haut Katanga have large urban centers while many other provinces (such as Mongala and Tshuapa) are nearly entirely rural, armed conflicts levels vary, and there are immense differences in provincial wealth [5]. This diversity of contextual characteristics relevant to RI strengthening across provinces is a limitation of our pooled analyses: comparing Mashako v. non-Mashako and MOU v. non-MOU provinces collectively may obfuscate relevant province-specific learnings. For example, the improvements in wealth-related inequalities in Tanganyika are relatively smaller than Haut Lomami's, but are still substantively larger than other conflict-affected provinces like Ituri – though even this comparison has limitations given the different scale of conflict within the provinces.

Another fundamental limitation of this analysis is the use of cross-sectional data; accordingly, we describe trends only and cannot directly attribute changes to Mashako or MOU approaches. In addition, when vaccinations cards were not available, the vaccination coverage estimates relied on maternal recall which may be biased and is generally less reliable than review of vaccination cards or health facility records [27,55,56]. Additionally, we used the MICS survey as our comparator time point before the implementation of the Mashako and MOUs and ECVs during implementation. Differences in the sampling approaches between MICS and ECVs, as well as between ECVs over time may have affected our estimates. [27] The MICS survey – representative at the provincial level – had a substantially smaller sample size (*N* = 4287 infants aged 12–23 months at the national level) compared to the ECV surveys (from *N* = 46,093 in 2020 to 46,990 in 2023) – representative below the province level, at the health zone level. This led to less precise provincial estimates from MICS, making it harder to detect statistically significant changes between the baseline (MICS 2017) and the implementation period (ECVs 2020–2023). [27] The 2020 ECV also covered fewer provinces than the other ECVs, making the pooled estimates by province groups in 2020 not directly comparable to other years [25]. Finally, potential limitations also emerged in the comparability of the annual ECV sampling strategies. Health areas were randomly sampled in 2020 (or 2021 for the eight provinces added in 2021). The same health areas were surveyed again in 2021 and 2022 (with few replacements). However, in 2023 the health areas were resampled, potentially leading to a less comparable sample of children. This makes it difficult to discern whether the estimated setbacks in 2023 may be partly attributable to changes in the sampling approach. Both MICS and ECV surveys may have also excluded more challenging to reach health areas. These health areas may be under-sampled and have both lower coverage and could contain less educated and less wealthy households, given the challenges in available infrastructure and services in rural DRC and the known link between distance to health services and vaccination uptake [57]. Ensuring the representativeness of ECVs moving forward is critical, particularly given they are meant to guide data-driven decision-making processes in real-time as part of the Mashako process [12].

An additional implication of our study is the persistence of vaccination inequalities. Although there were improvements in reducing inequalities, particularly in wealth-related inequalities between 2017 and 2020–2021, the pace of progress seems to have slowed in 2022–2023. The initial aims of the Mashako 1.0 implementation focused broadly on health systems strengthening [12]. Achieving further gains in reducing inequalities may require more explicitly equity-focused efforts in future Mashako activities. Relevant pro-equity strategies may include a stronger focus on community-focused demand-creation strategies and strengthening non-facility-based vaccination delivery in hard-to-reach areas [52]. In the DRC, a prior analysis found that access barriers were more strongly associated with under-vaccination status than vaccine hesitancy, while social factors were more strongly linked to complete non-vaccination [11]. Existing work in the DRC such as drone vaccination and geospatial microplanning, may address challenges in demand generation and hard-to-reach populations, yet require further evaluation of impact and scalability. [58,59] Finally, given our hypothesis that early MOU and Mashako gains may have been driven by last-mile cold chain investments, these improvements will only be maintained with appropriate investments in maintaining existing vehicles and cold chain equipment. This may help explain the decline in coverage and rising inequality seen in 2022–23. Early cold chain investments likely supported initial gains among harder-to-reach groups, but a lack of sustained investment may have led to subsequent setbacks. Finally, given the stagnation in maternal education-related inequalities, gender transformative and social norms-related strategies may be especially relevant [52]. Importantly, for such interventions to be appropriately designed and targeted, monitoring inequalities in vaccination coverage should become a part of DRC's routine monitoring of RI systems.

This study describes persistent wealth and educational vaccination inequalities in the DRC. Progress has been made in the last five years, including in provinces where RI strengthening initiatives are being implemented. However, stagnation in both inequality improvements and vaccination coverage during most recent years points to a critical need to routinely assess inequalities and incorporate equity-specific approaches in RI strengthening initiatives moving forward.

## Authors' contributions

CA and EL conceptualized the analysis. EL and CA analysed the data. EL and CA led the writing of the paper. BKM, JKNM, PSLD, SN, TH, and WM contributed to the writing of the paper.

## CRediT authorship contribution statement

**Elise Lankiewicz:** Conceptualization, Writing – original draft, Formal analysis. **Junias Kabele Ngoy Mpemba:** Writing – review & editing, Data curation. **Paul Samson Lusamba Dikassa:** Writing – review & editing. **Viviane Mayala Masiala:** Writing – review & editing. **Benito Kazenza Maykondo:** Writing – review & editing. **Trad Hatton:** Writing – review & editing. **Saira Nawaz:** Writing – review & editing. **Wolfgang Munar:** Writing – review & editing. **Catherine Arsenault:** Writing – review & editing, Writing – original draft, Formal analysis, Conceptualization.

## Declaration of competing interest

The authors declare the following financial interests/personal relationships which may be considered as potential competing interests: Catherine Arsenault reports financial support was provided by Bill & Melinda Gates Foundation. If there are other authors, they declare that they have no known competing financial interests or personal relationships that could have appeared to influence the work reported in this paper.

## Data Availability

The data sets are available upon request from the dataset owners at Kinshasa School of Public Health.

## Appendix A Supplementary data

Supplementary material - MCV coverage and inequalities Image 1
