# Supplementary material for: Trends in vaccination coverage and equity in the Democratic Republic of the Congo from 2017 to 2023
Source: Vaccine. 2025 Aug 30;62:None. doi: 10.1016/j.vaccine.2025.127609 (PMC12447091; doi:10.1016/j.vaccine.2025.127609)
Supplement: Supplementary file 1 — Supplementary material - MCV coverage and inequalities [file mmc1.docx]

| Appendix Table 1. Penta3 coverage estimates at the national level and in Mashako 1.0 and non-Mashako 1.0 provinces from 2017 to 2023. | | | | | |
| --- | --- | --- | --- | --- | --- |
|  | 2017 | 2020^a^ | 2021 | 2022 | 2023 |
| National | 47.68 (43.45, 51.92) | 67.24 (66.16, 68.32) | 60.27 (59.19, 61.36) | 61.27 (60.19, 62.36) | 57.61 (56.57, 58.65) |
| Non-Mashako | 49.83 (44.45, 55.20) | 63.21 (61.80, 64.62) | 56.48 (55.04, 57.92) | 56.74 (55.35, 58.13) | 52.46 (51.09, 53.83) |
| Mashako 1.0^b^ | 44.62 (38.05, 51.19) | 70.85 (69.25, 72.45) | 65.34 (63.71, 66.96) | 67.26 (65.62, 68.91) | 64.08 (62.45, 65.71) |
| MOU 1.0 Mashako^c^ | 32.20 (20.64, 43.77) | 71.16 (67.16, 75.16) | 69.11 (64.39, 73.84) | 78.15 (73.99, 82.32) | 69.83 (65.59, 74.06) |
| Non-MOU 1.0 Mashako^d^ | 46.29 (39.02, 53.56) | 70.79 (69.04, 72.53) | 64.58 (62.88, 66.28) | 64.58 (62.79, 66.37) | 62.95 (61.19, 64.71) |
| ^a^ In 2020 only 18 of 26 provinces were surveyed compared to all 26 in all other years of data.  ^b^Mashako 1.0 provinces include those included in the original implementation of the Mashako Plan: Mongala, Tshuapa, Haut Katanga, Tanganyika, Haut Lomami, Ituri, Kinshasa, Kwilu, and Kasaï. The non-Mashako group includes all other provinces in the country.  ^c^The MOU 1.0 Mashako group includes Haut Lomami and Tanganyika.  ^d^The non-MOU 1.0 group includes: Mongala, Tshuapa, Haut Katanga, Ituri, Kinshasa, Kwilu, and Kasaï. | | | | | |

| Appendix Table 2. MCV coverage estimates at the national level and in Mashako 1.0 and non-Mashako 1.0 provinces from 2017 to 2023. | | | | | |
| --- | --- | --- | --- | --- | --- |
|  | 2017 | 2020^a^ | 2021 | 2022 | 2023 |
| National | 58.87 (55.08, 62.66) | 68.54 (67.58, 69.51) | 55.50 (54.41, 56.59) | 56.08 (54.98, 57.17) | 52.22 (51.17, 53.27) |
| Non-Mashako | 58.17 (53.64, 62.70) | 63.13 (61.76, 64.50) | 49.36 (47.91, 50.80) | 50.46 (49.07, 51.84) | 46.02 (44.64, 47.39) |
| Mashako 1.0^b^ | 59.87 (53.25, 66.49) | 73.40 (72.07, 74.72) | 63.69 (62.11, 65.27) | 63.51 (61.82, 65.20) | 60.01 (58.39, 61.63) |
| MOU 1.0 Mashako^c^ | 43.37 (31.31, 55.43) | 76.60 (73.47, 79.72) | 63.72 (58.92, 68.52) | 70.18 (65.21, 75.16) | 60.53 (56.49, 64.56) |
| Non-MOU 1.0 Mashako^d^ | 62.08 (54.74, 69.42) | 72.75 (71.28, 74.21) | 63.69 (62.05, 65.32) | 61.86 (60.12, 63.60) | 59.91 (58.14, 61.68) |
| ^a^ In 2020 only 18 of 26 provinces were surveyed compared to all 26 in all other years of data.  ^b^Mashako 1.0 provinces include those included in the original implementation of the Mashako Plan: Mongala, Tshuapa, Haut Katanga, Tanganyika, Haut Lomami, Ituri, Kinshasa, Kwilu, and Kasaï. The non-Mashako group includes all other provinces in the country.  ^c^The MOU 1.0 Mashako group includes Haut Lomami and Tanganyika.  ^d^The non-MOU 1.0 group includes: Mongala, Tshuapa, Haut Katanga, Ituri, Kinshasa, Kwilu, and Kasaï. | | | | | |

Mashako 1.0

(No Kinshasa)

National

Kinshasa

Non-Mashako 1.0

Estimated Survey Values

Note: In 2020 only 18 of 26 provinces were surveyed compared to all 26 in all other years of data.

Mashako 1.0 provinces include those included in the original implementation of the Mashako Plan except Kinshasa: Mongala, Tshuapa, Haut Katanga, Tanganyika, Haut Lomami, Ituri, Kwilu, and Kasaï. The non-Mashako group includes all other provinces in the country besides Kinshasa.


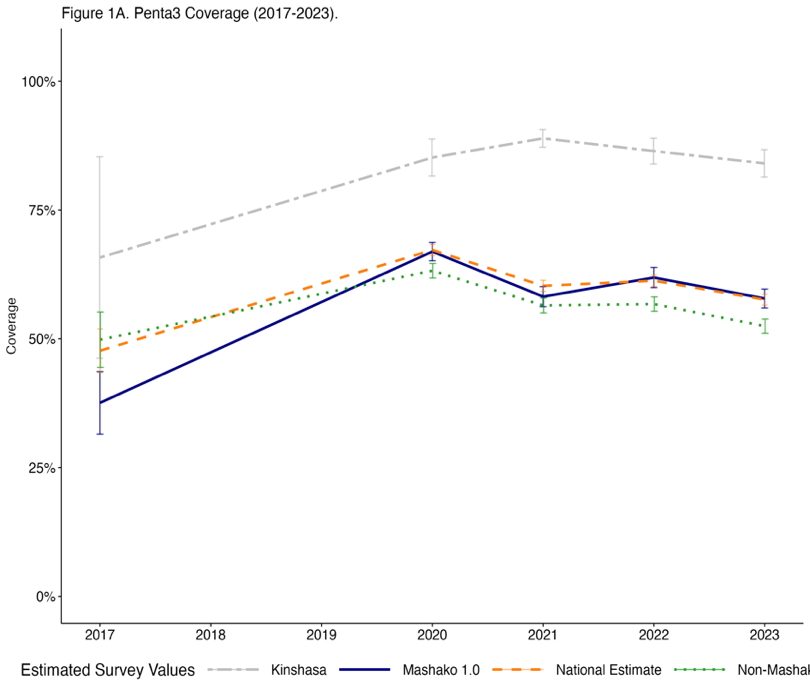


**Appendix Figure 1. Penta3 coverage estimates at the national level, Mashako 1.0 (no Kinshasa), non-Mashako 1.0 provinces, and Kinshasa from 2017 to 2023.**

**Appendix Figure 2. Penta3 coverage estimates within Mashako provinces (2017-2023), MOU 1.0, Kinshasa, other non-MOU 1.0.**


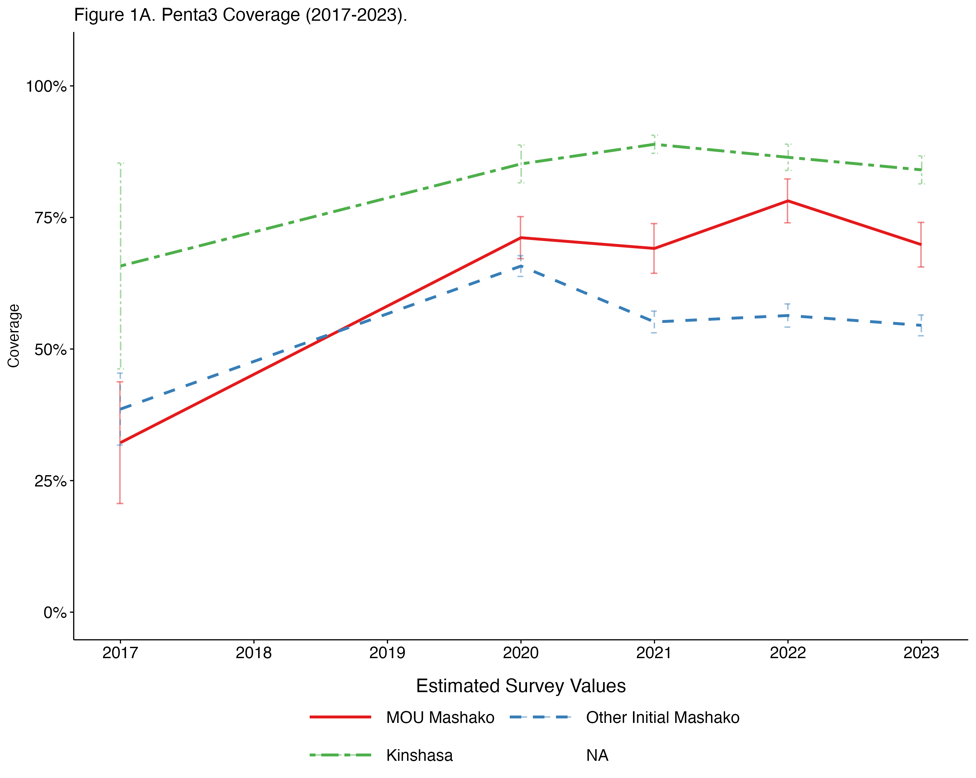


MOU 1.0

Other Initial Mashako 1.0

Kinshasa

Note: In 2020 only 18 of 26 provinces were surveyed compared to all 26 in all other years of data.

The MOU 1.0 Mashako group includes Haut Lomami and Tanganyika. The Other Initial Mashako group includes: Mongala, Tshuapa, Haut Katanga, Ituri, Kwilu, and Kasaï.


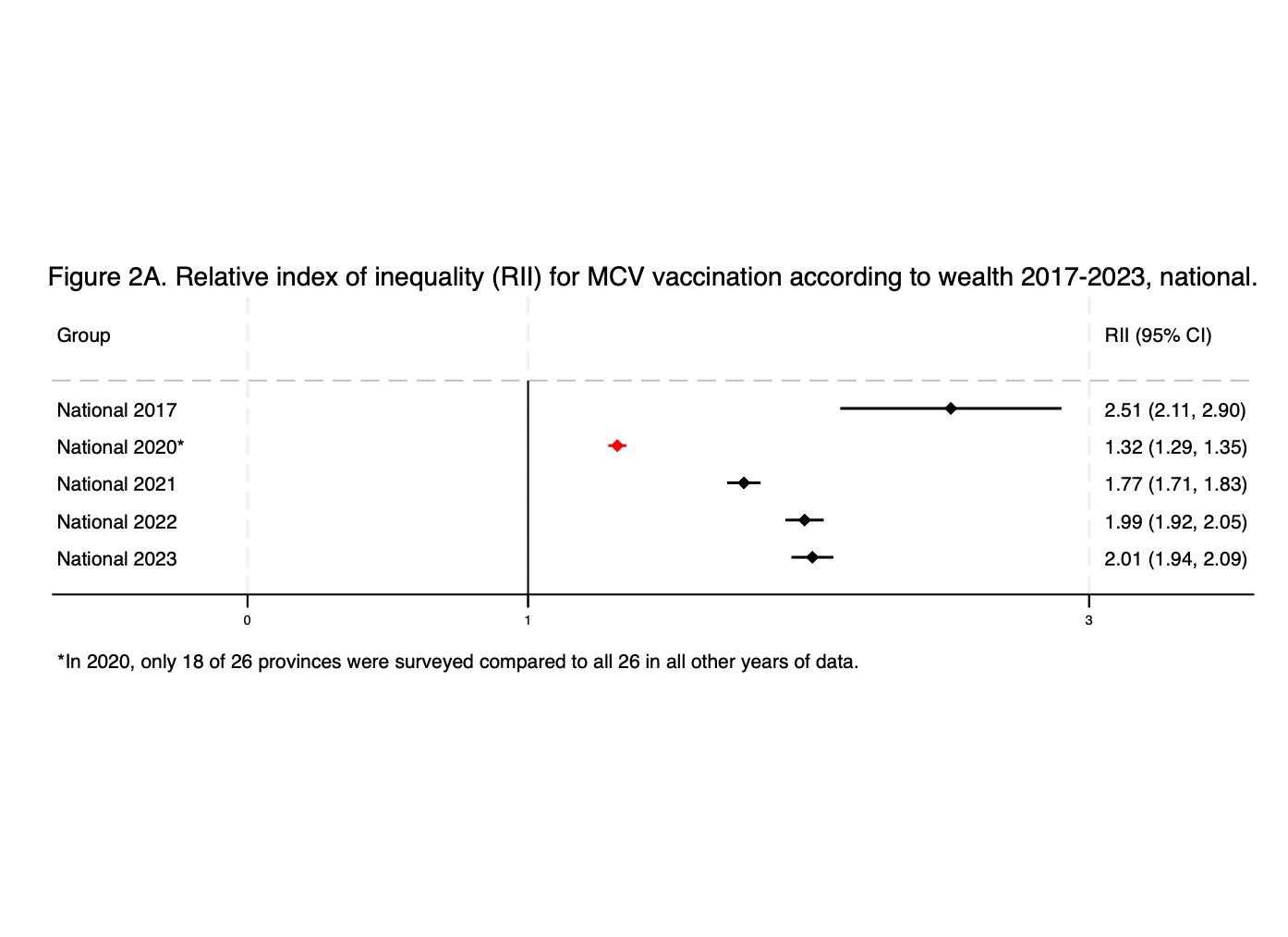


**Appendix Figure 3A. National-level wealth-related relative index of inequality (RII) in MCV vaccination (2017-2023)**


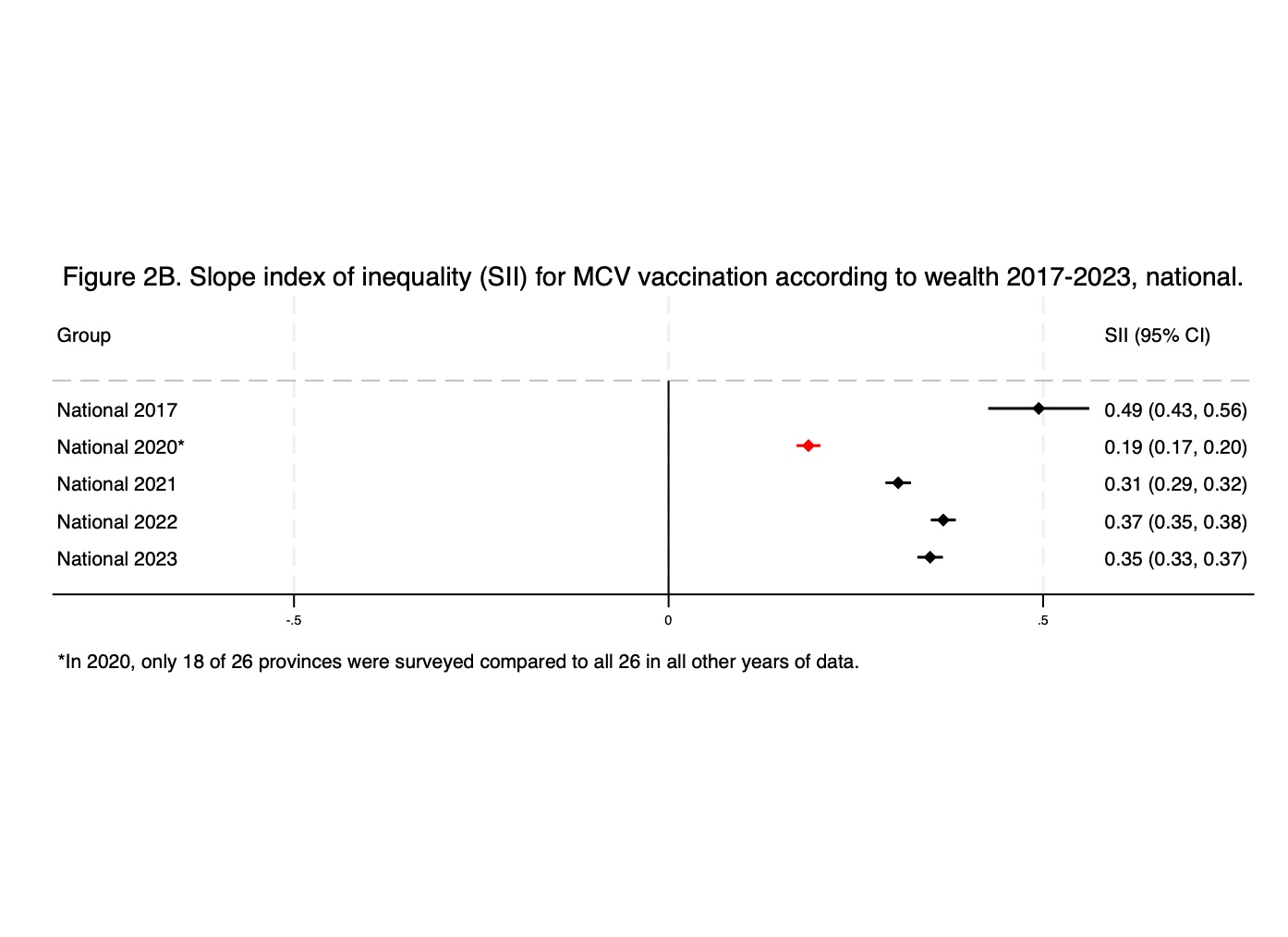


**Appendix Figure 3B. National-level wealth-related slope index of inequality (SII) in MCV vaccination (2017-2023)**


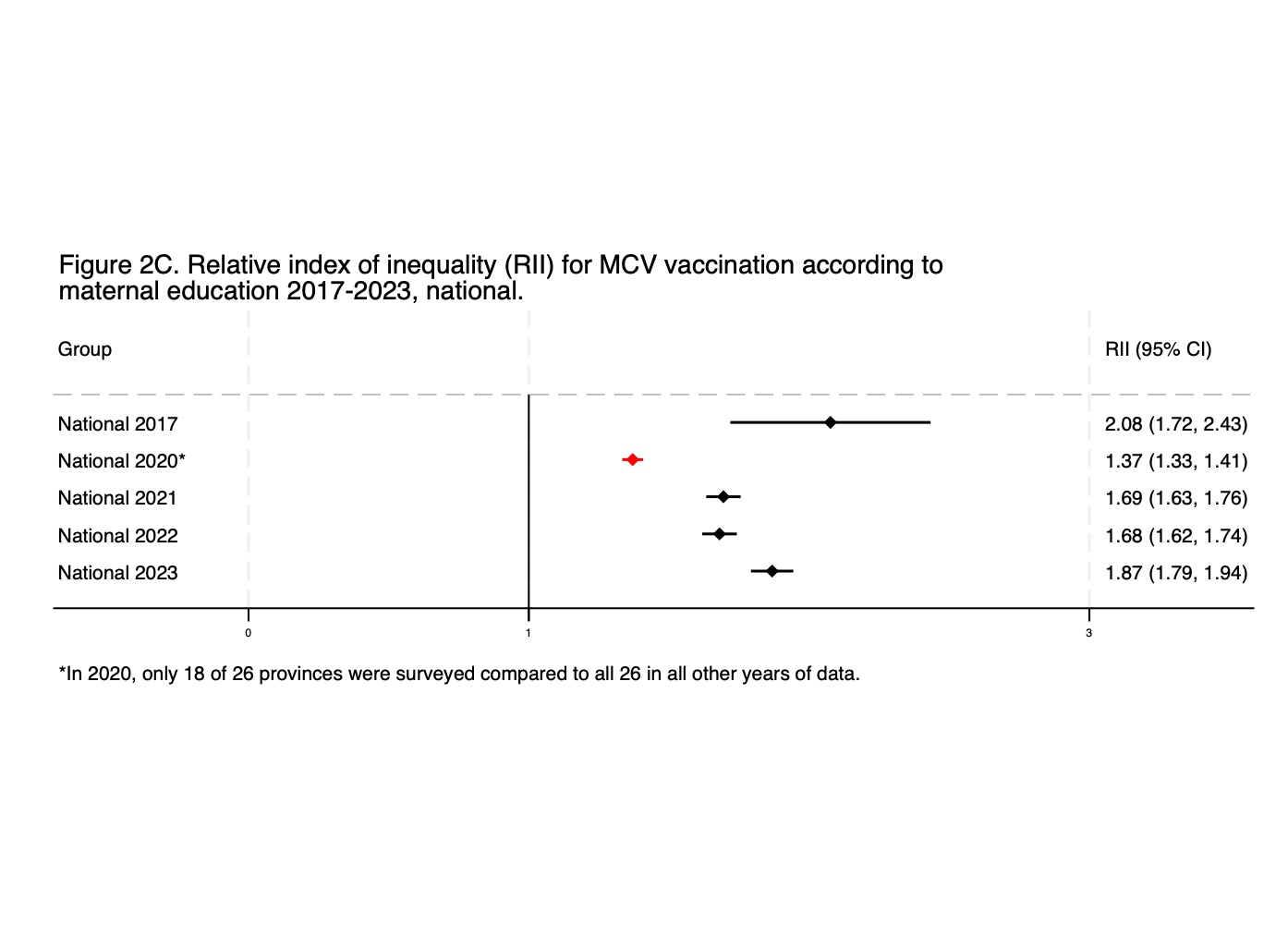


**Appendix Figure 3C. National-level education-related relative index of inequality (RII) in MCV vaccination (2017-2023)**


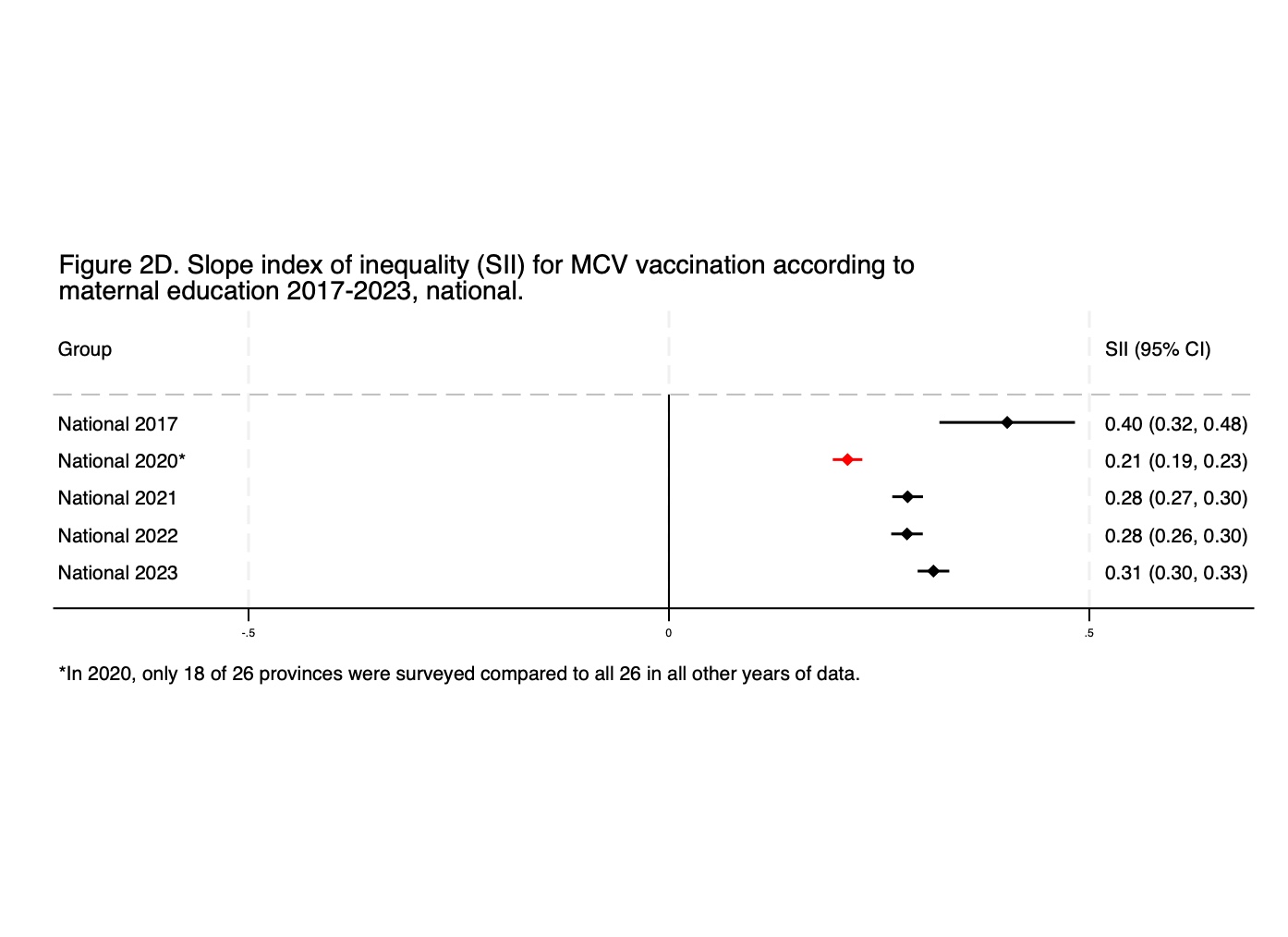


**Appendix Figure 3D. National-level education-related slope index of inequality (SII) in MCV vaccination (2017-2023)**

**Appendix Figure 4A. Pooled wealth-related relative index of inequality (RII) in MCV vaccination (2017-2023) in Mashako 1.0^&^ vs. non-Mashako provinces**


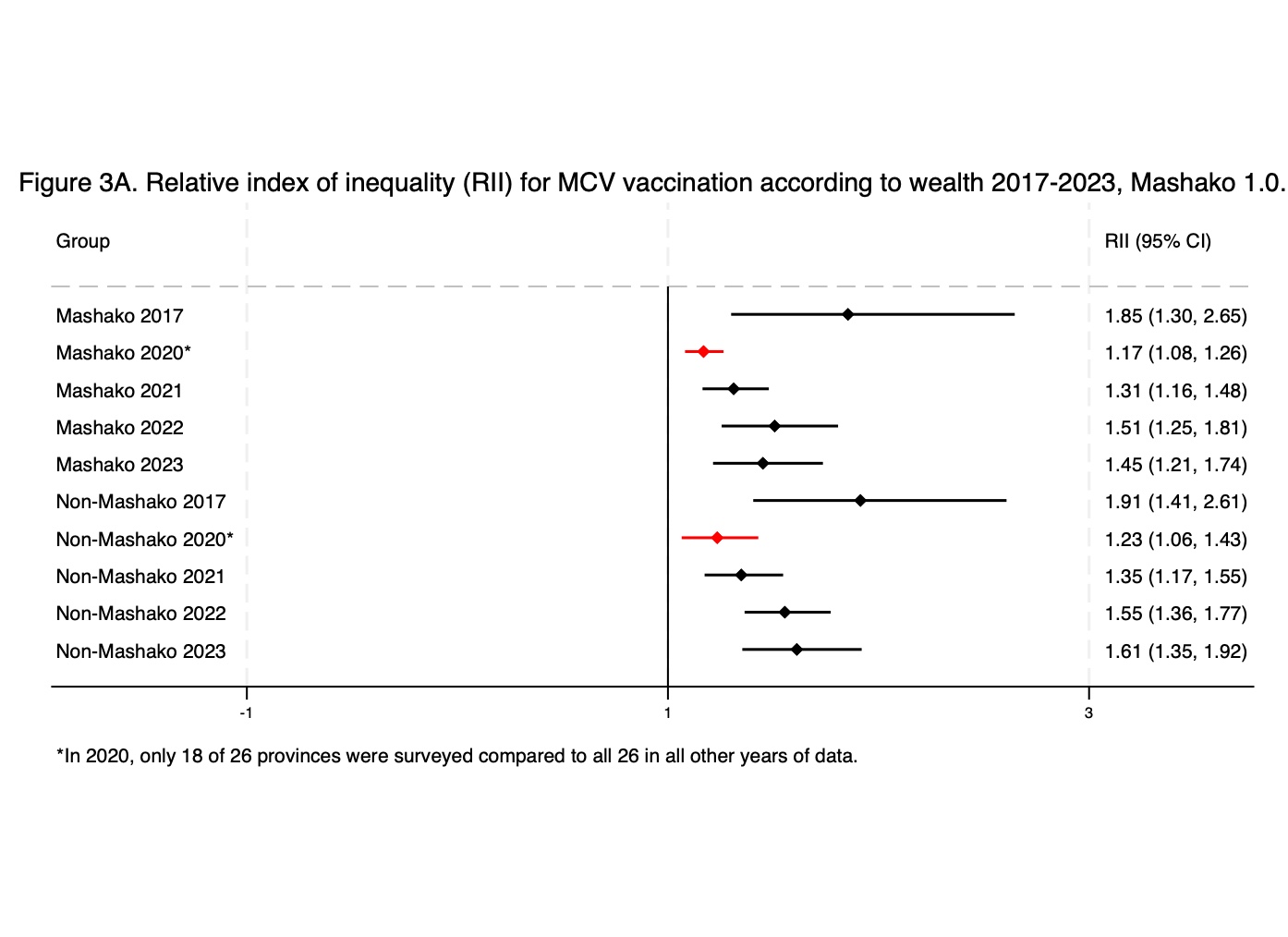


* In 2020 only 18 of 26 provinces were surveyed compared to all 26 in all other years of data.

^&^  Mashako provinces include those included in the original implementation of the Mashako Plan 1.0: Mongala, Tshuapa, Haut Katanga, Tanganyika, Haut Lomami, Ituri, Kinshasa, Kwilu, and Kasaï.The non-Mashako group includes all other provinces in the country.


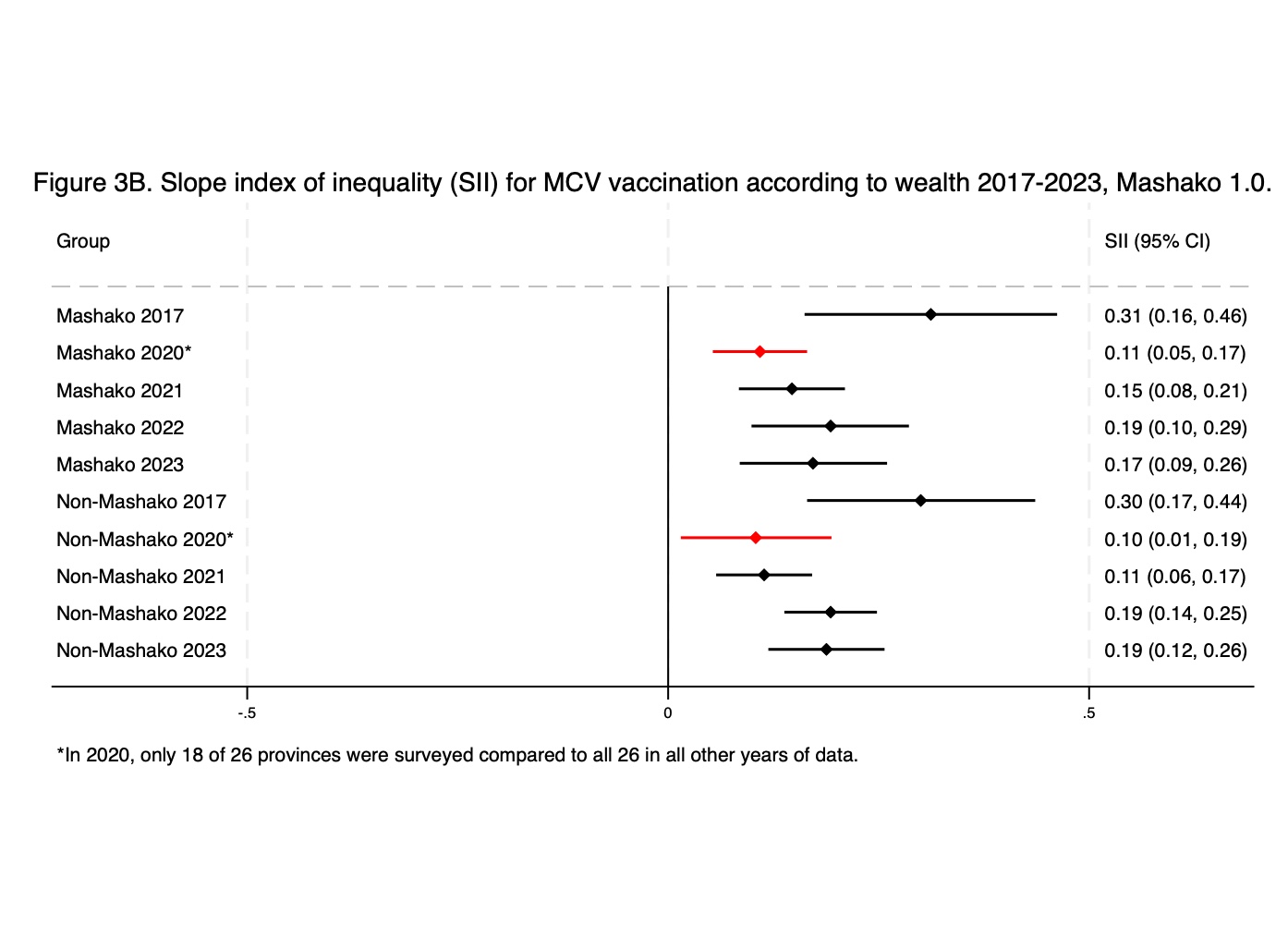


* In 2020 only 18 of 26 provinces were surveyed compared to all 26 in all other years of data.

^&^  Mashako provinces include those included in the original implementation of the Mashako Plan 1.0: Mongala, Tshuapa, Haut Katanga, Tanganyika, Haut Lomami, Ituri, Kinshasa, Kwilu, and Kasaï.The non-Mashako group includes all other provinces in the country.

**Appendix Figure 4B. Pooled wealth-related slope index of inequality (SII) in MCV vaccination (2017-2023) in Mashako 1.0^&^ vs. Non-Mashako provinces**


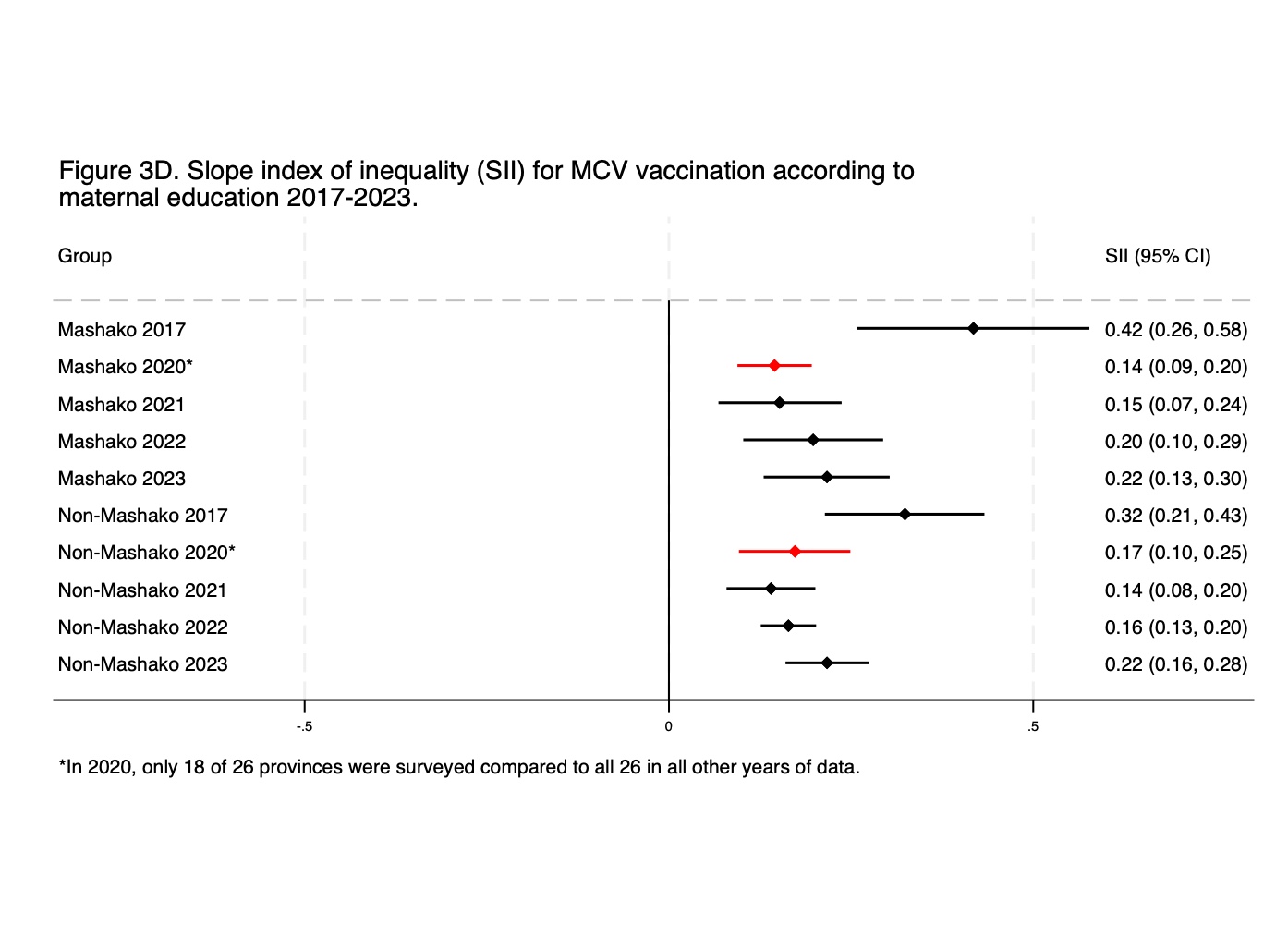


**Appendix Figure 4D. Pooled educated-related slope index of inequality (SII) in MCV vaccination according (2017-2023) in Mashako 1.0^&^ vs. Non-Mashako provinces**

* In 2020 only 18 of 26 provinces were surveyed compared to all 26 in all other years of data.

^&^  Mashako provinces include those included in the original implementation of the Mashako Plan 1.0: Mongala, Tshuapa, Haut Katanga, Tanganyika, Haut Lomami, Ituri, Kinshasa, Kwilu, and Kasaï.The non-Mashako group includes all other provinces in the country.


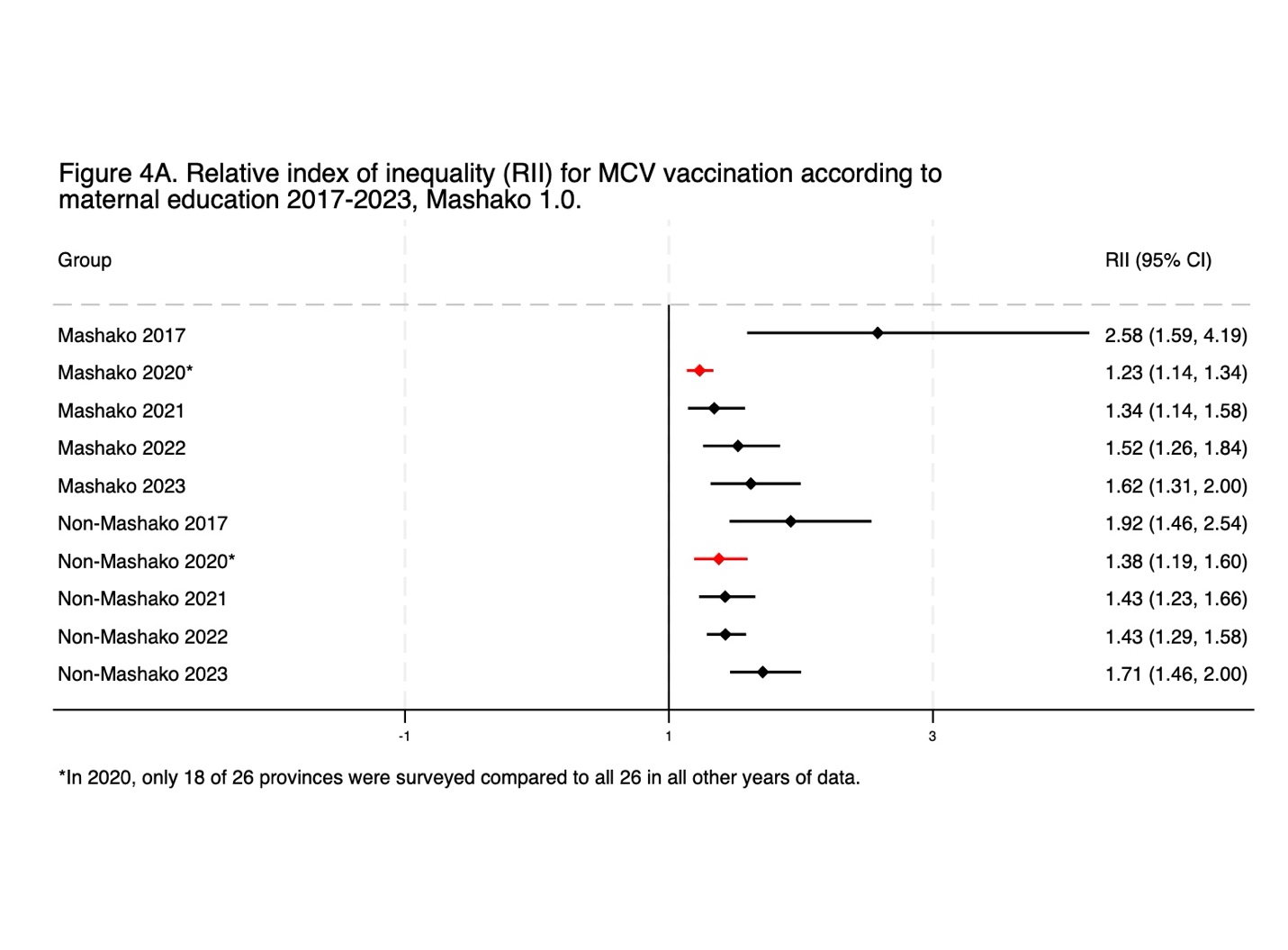


**Appendix Figure 4C. Pooled education-related relative index of inequality (RII) in MCV vaccination (2017-2023) in Mashako 1.0^&^ vs. Non-Mashako provinces**

* In 2020 only 18 of 26 provinces were surveyed compared to all 26 in all other years of data.

^&^  Mashako provinces include those included in the original implementation of the Mashako Plan 1.0: Mongala, Tshuapa, Haut Katanga, Tanganyika, Haut Lomami, Ituri, Kinshasa, Kwilu, and Kasaï.The non-Mashako group includes all other provinces in the country.

**Appendix Figure 5A. Wealth-related relative index of inequality (RII) in MCV vaccination, pre-MOU (2017) and endline-MOU 1.0 (2022)**


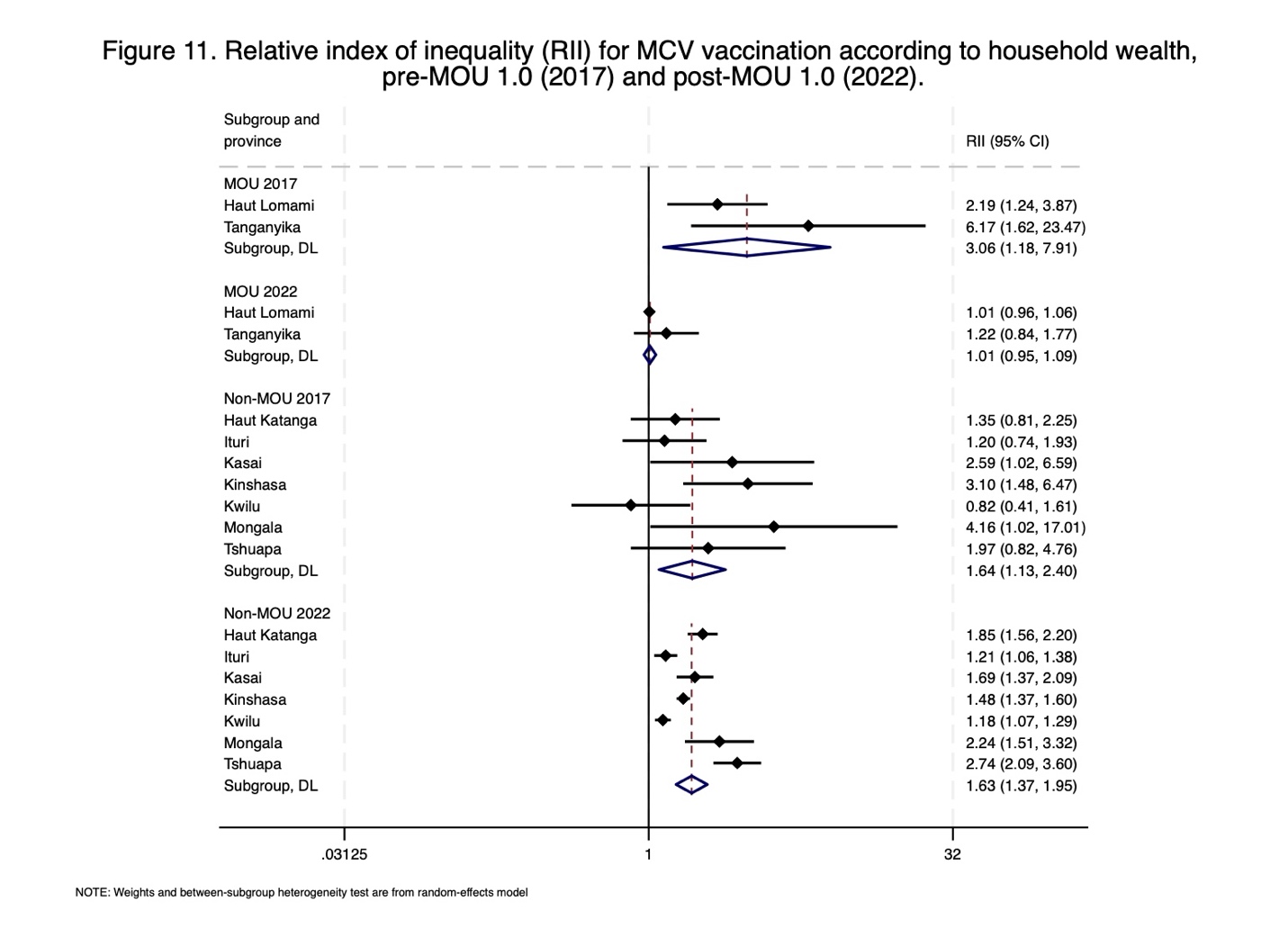


**Appendix Figure 5B. Wealth-related slope index of inequality (SII) in MCV vaccination, pre-MOU 1.0 (2017) and endline-MOU 1.0 (2022)**


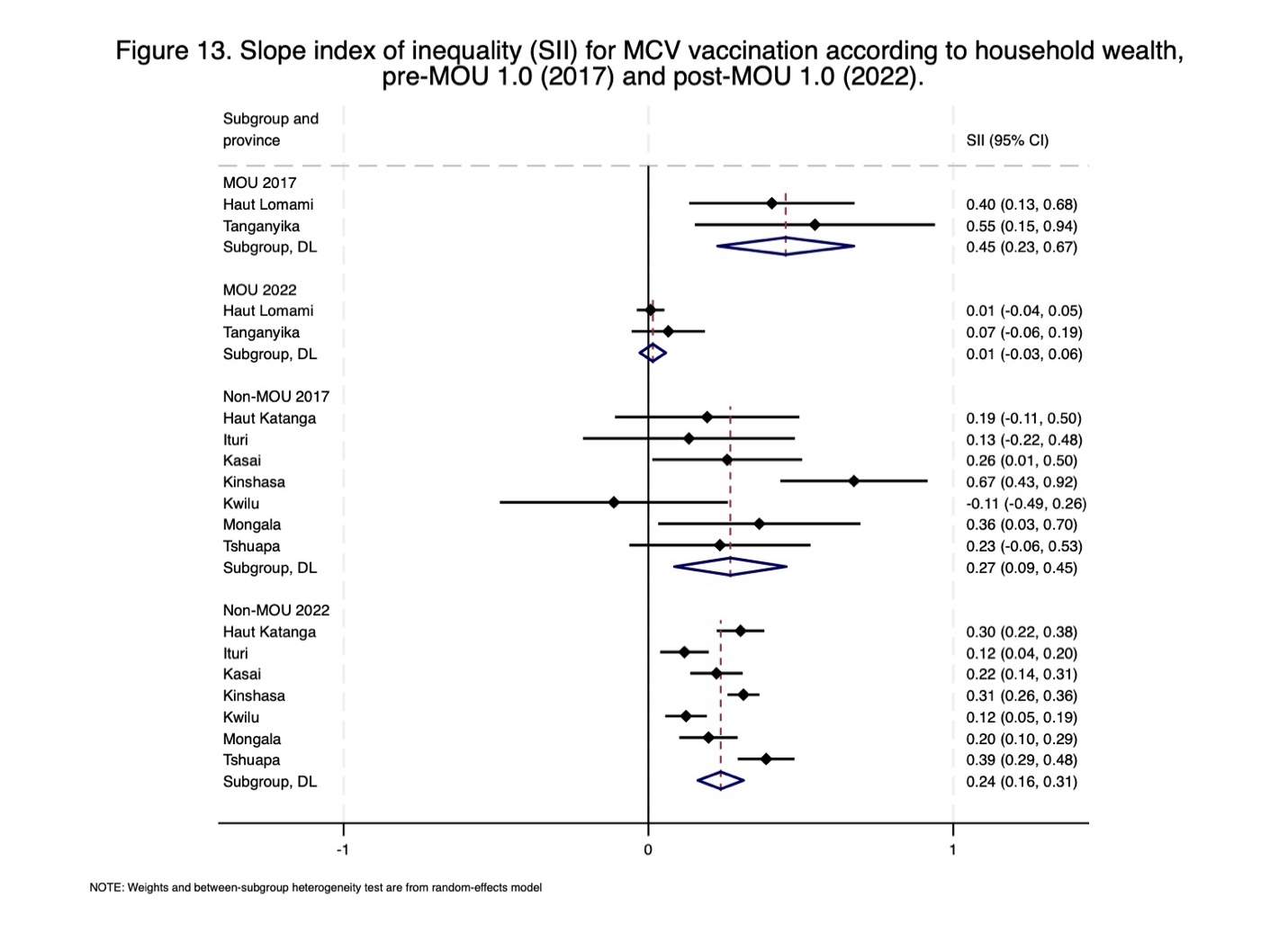


**Figure 5D. Education-related slope index of inequality (SII) in MCV, pre-MOU 1.0 (2017) and endline-MOU 1.0 (2022)**


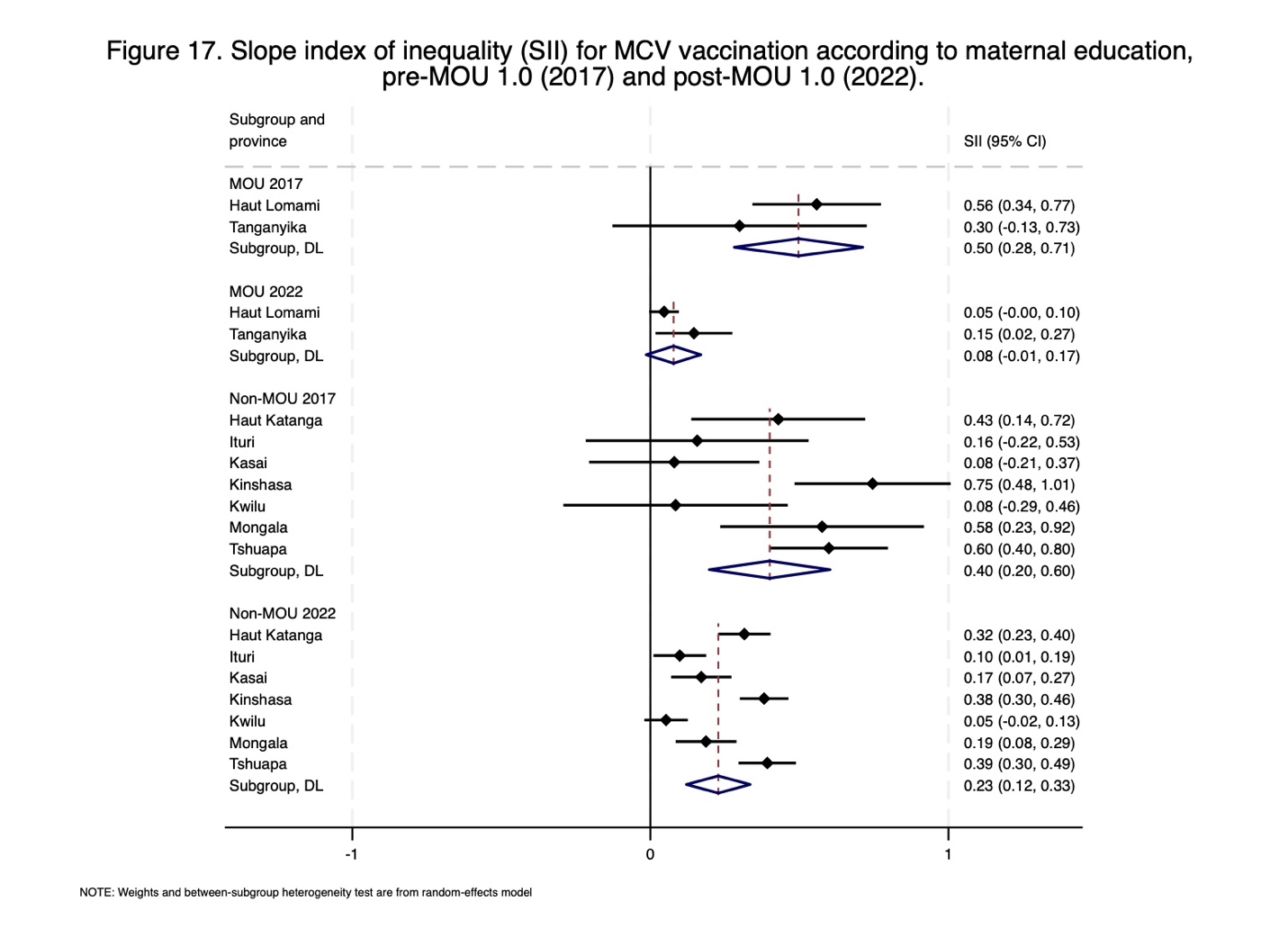


**Appendix Figure 5C. Education-related relative index of inequality (RII) in MCV, pre-MOU 1.0 (2017) and endline-MOU 1.0 (2022)**


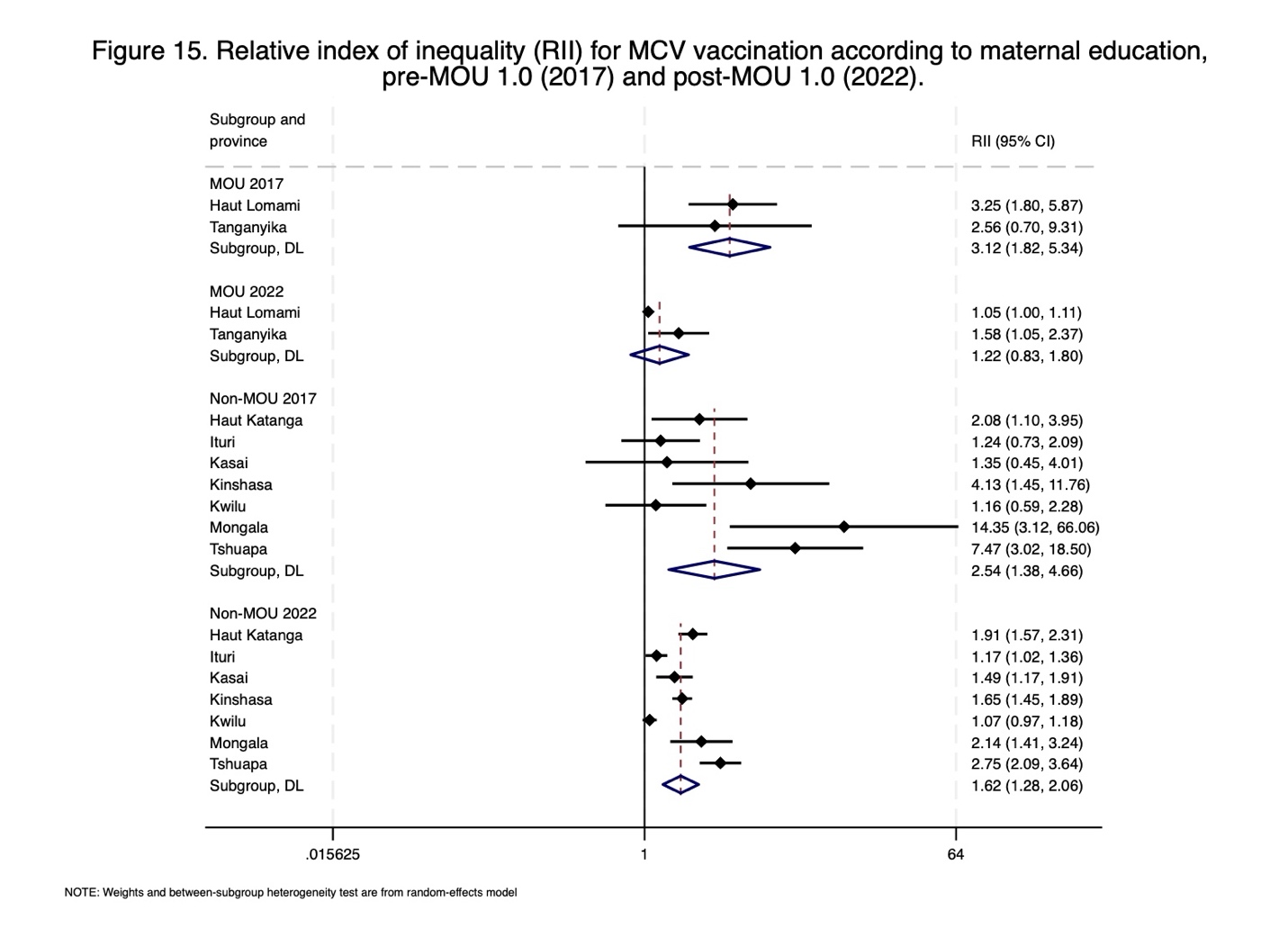


| Appendix Table 3. Change in MCV inequality during the Mashako 1.0^a^ period 2017 – 2021 (95% CI) | | | | | | | | | | | | |
| --- | --- | --- | --- | --- | --- | --- | --- | --- | --- | --- | --- | --- |
|  | | **RII 2017 (CI)** | | **RII 2021 (CI)** | | **RII Change (CI)** | | **SII 2017 (CI)** | | **SII 2021 (CI)** | | **SII Change (CI)** |
| Wealth | | | | | | | | | | | | |
| Mashako | 1.85 (2.65, 1.3) | | 1.31 (1.48, 1.16) | | -0.54 (-1.23, 0.15) | | 0.31 (0.46, 0.16) | | 0.15 (0.21, 0.08) | | -0.16 (-0.33, 0)* | |
| Non-Mashako | 1.91 (2.61, 1.41) | | 1.35 (1.55, 1.17) | | -0.57 (-1.2, 0.06) | | 0.3 (0.44, 0.16) | | 0.11 (0.17, 0.06) | | -0.19 (-0.33, -0.04)* | |
| Difference, Mashako - Non-Mashako |  | |  | | 0.02 (-0.91, 0.96) | |  | |  | | 0.02 (-0.20, 0.24) | |
| Education | | | | | | | | | | | | |
| Mashako | 2.58 (4.19, 1.59) | | 1.34 (1.58, 1.14) | | -1.24 (-2.55, 0.08) | | 0.42 (0.58, 0.26) | | 0.15 (0.24, 0.07) | | -0.27 (-0.45, -0.09)* | |
| Non-Mashako | 1.92 (2.54, 1.46) | | 1.43 (1.66, 1.23) | | -0.5 (-1.08, 0.08) | | 0.32 (0.43, 0.21) | | 0.14 (0.2, 0.08) | | -0.18 (-0.31, -0.06)* | |
| Difference, Mashako - Non-Mashako |  | |  | | -0.74 (-2.18, 0.69) | |  | |  | | -0.08 (-0.30, 0.14) | |
| ^a^Mashako provinces include those included in the original implementation of the Mashako Plan 1.0: Mongala, Tshuapa, Haut Katanga, Ituri, Kinshasa, Kwilu, and Kasaï. The non-Mashako group includes all other provinces in the country. | | | | | | | | | | | | |

| Appendix Table 4. Change in MCV inequality during the MOU 1.0^a^ period 2017 – 2022 (95% CI) | | | | | | | | | | | | |
| --- | --- | --- | --- | --- | --- | --- | --- | --- | --- | --- | --- | --- |
|  | | **RII 2017 (CI)** | | **RII 2022 (CI)** | | **RII Change (CI)** | | **SII 2017 (CI)** | | **SII 2022 (CI)** | | **SII Change (CI)** |
| Wealth | | | | | | | | | | | | |
| MOU | 3.06 (7.91, 1.18) | | 1.01 (1.09, 0.95) | | -2.05 (-5.42, 1.32) | | 0.45 (0.67, 0.23) | | 0.01 (0.06, -0.03) | | -0.44 (-0.66, -0.22)* | |
| Non-MOU | 1.64 (2.40, 1.12) | | 1.63 (1.95, 1.37) | | -0.01 (-0.72, 0.70) | | 0.27 (0.45, 0.09) | | 0.24 (0.31, 0.16) | | -0.03 (-0.23, 0.17) | |
| *Difference, MOU - Non-MOU* |  | |  | | -2.03 (-5.47, 1.4) | |  | |  | | -0.4 (-0.71, -0.1) | |
| Education | | | | | | | | | | | | |
| MOU | 3.12 (5.34, 1.82) | | 1.22 (1.79, 0.83) | | -1.90 (-3.72, -0.08)* | | 0.50 (0.71, 0.28) | | 0.08 (0.17, -0.01) | | -0.42 (-0.66, -0.18)* | |
| Non-MOU | 2.54 (4.66, 1.39) | | 1.62 (2.06, 1.28) | | -0.92 (-2.61, 0.77) | | 0.40 (0.60, 0.20) | | 0.23 (0.34, 0.12) | | -0.17 (-0.39, 0.05) | |
| *Difference, MOU - Non-MOU* |  | |  | | -0.98 (-3.46, 1.5) | |  | |  | | -0.25 (-0.57, 0.08) | |
| ^a^The MOU group includes Haut Lomami and Tanganyika. The non-MOU group includes Haut Katanga, Ituri, Kasaï, Kinshasa, Kwilu, Mongala, and Tshuapa.  Note: * indicates significance at p<0.05. | | | | | | | | | | | | |
